# Receptor usage of Syncytin-1: ASCT2, but not ASCT1, is a functional receptor and effector of cell fusion in the human placenta

**DOI:** 10.1073/pnas.2407519121

**Published:** 2024-10-21

**Authors:** Kryštof Štafl, Martin Trávníček, Anna Janovská, Dana Kučerová, Ľubomíra Pecnová, Zhiqi Yang, Vladimír Stepanec, Lukáš Jech, Madhuri S. Salker, Jiří Hejnar, Kateřina Trejbalová

**Affiliations:** ^a^Laboratory of Viral and Cellular Genetics, Institute of Molecular Genetics of the Czech Academy of Sciences, Vídeňská 14220, Czech Republic; ^b^CZ-OpenScreen National Infrastructure for Chemical Biology, Institute of Molecular Genetics of the Czech Academy of Sciences, Vídeňská 14220, Czech Republic; ^c^Department of Women's Health, University of Tübingen, Tübingen 72076, Germany

**Keywords:** Syncytin-1, viral receptor, cell-to-cell fusion, placenta, endogenous retrovirus

## Abstract

Syncytin-1, a human fusogenic protein of retroviral origin, is crucial for placental syncytiotrophoblast formation. To mediate cell-to-cell fusion, Syncytin-1 requires specific interaction with its cognate receptor. Two trimeric transmembrane proteins, Alanine, Serine, Cysteine Transporters 1 and 2 (ASCT1 and ASCT2), were suggested and widely accepted as Syncytin-1 cellular receptors. To quantitatively assess the individual contributions of human ASCT1 and ASCT2 to the fusogenic activity of Syncytin-1, we developed a model system where the *ASCT1* and *ASCT2* double knockout was rescued by ectopic expression of either ASCT1 or ASCT2. We demonstrated that ASCT2 was required for Syncytin-1 binding, cellular entry, and cell-to-cell fusion, while ASCT1 was not involved in this receptor interaction. We experimentally validated the ASCT1–ASCT2 heterotrimers as a possible explanation for the previous misidentification of ASCT1 as a receptor for Syncytin-1. This redefinition of receptor specificity is important for proper understanding of Syncytin-1 function in normal and pathological pregnancy.

## Significance

Our work addressed the receptor usage of Syncytin-1, essential for controlling the fusion of human placental cells during pregnancy. We identified human Alanine, Serine, Cysteine Transporter (ASCT) 2 as the sole receptor for Syncytin-1. Contrary to previous studies, we assume that human ASCT1 does not interact with Syncytin-1, either inside or outside the placenta. We detected a heterotrimeric complementation between ASCT2 and ASCT1 molecules, and we point out that this transcomplementation led to the misassignment of the ASCT1 receptor role. We further showed that certain viruses in the RDR interference group, which infect different hosts, can utilize not only human or orthologous ASCT2 but also ASCT1. Primarily, our findings resolve human ASCT2 as a single-specific receptor for Syncytin-1-dependent cell-to-cell fusion.

Formation of the hemochorial placenta is essential for successful human pregnancy and fetal health. Derived from extraembryonic tissues, the placenta rapidly develops during the first weeks of gestation dynamically changing its structure and function (1–4). Failure in optimal placental formation can compromise fetal growth and development. Abnormal placentation can lead to adverse pregnancy outcomes including preeclampsia and growth restriction resulting in considerable maternal and/or infant mortality and morbidity rates (5–7). Despite its critical role in reproduction, placental morphogenesis remains poorly understood.

One of the key processes in placental development is the fusion of trophoblast cells into multinucleated cells termed syncytiotrophoblasts. This fusion process is mediated by the proteins Syncytin-1 and Syncytin-2, which are encoded by retroviral envelope genes that have been exapted for their role in placentation (8). Syncytin-1, an envelope glycoprotein of the human endogenous retrovirus HERV-W, permitted an ancient retrovirus to infect the host cells of our ancestors. To turn fusogenic *syncytin*-*1* into a regular human gene, its expression had to be strictly regulated on both transcriptional and posttranscriptional levels (9–17). Under pathological conditions, *syncytin*-*1* mRNA was found in germ cell tumors (15, 18). The findings further indicated its expression in the astrocytes, microglia, and immune cells of multiple sclerosis patients (19–21). In healthy individuals, Syncytin-1 expression is restricted specifically to the placenta, where it fuses adjacent cells and mediates syncytiotrophoblast formation (9–17).

Triggering the fusion of two cellular membranes requires interaction with its cognate receptor on the surface of the neighboring cell (9, 22). Two receptors for Syncytin-1 have been proposed: ASCT2 (Alanine, Serine, Cysteine Transporter 2, SLC1A5) and later ASCT1 (SLC1A4) (22, 23). Both receptors are sodium-dependent neutral amino acid transporters with resolved cryoelectron microscopy structures (24–26). Importantly, both proteins assemble into trimers. ASCT1 preferentially transports alanine, serine, and cysteine (27). It is involved in brain metabolism and regulates extracellular serine levels, affecting synaptic plasticity and neurotransmission (28, 29). In addition to alanine and serine, ASCT2 transports threonine and especially glutamine (30). Therefore, ASCT2 is involved in various physiological and pathological processes, provides glutamine for protein synthesis and energy metabolism, and promotes ammonia detoxification (31).

Under physiological conditions, the tissue localization of ASCT1, ASCT2, and Syncytin-1 differs. While Syncytin-1 expression is limited to placental cytotrophoblast and syncytiotrophoblast (9–17), both of its proposed receptors are widely expressed. *ASCT1* mRNA was detected in all tissues tested, including the placenta, but predominantly in the brain, skeletal muscles, and pancreas (27, 32). *ASCT2* mRNA was found in the placenta, lungs, large intestine, kidneys, skeletal muscles, testes, adipose tissue, and pancreas (30). However, at the protein level, ASCT1 was not detected in the placenta, in contrast to the highly abundant ASCT2 (33). This raises the pertinent point of how ASCT1 participates in human placenta morphogenesis and what is the contribution of each receptor to Syncytin-1-mediated cell-to-cell fusion.

Human ASCT2 was described as the receptor not only for Syncytin-1 but also for some other retroviruses, such as feline endogenous RD114, baboon endogenous retrovirus (BaEV), and Simian type D retrovirus 2 (SRV-2) (34–36). These viruses form a large RD114-and-D-type-retrovirus (RDR) interference group, which also contains squirrel monkey retrovirus (SMRV) and Mason-Pfizer monkey virus (MPMV) (37, 38). Although most of these viruses are endogenous, some are still circulating in exogenous form (39) and can potentially enter human cells via the human ASCT2 receptor. Previous research also showed that envelope glycoproteins of BaEV and RD114, but not SRV-2, interact with human ASCT1 (36). Thus, although they belong to the same interference group, the envelope glycoproteins of RDR viruses are not identical, and their receptor recognition and interaction interfaces may differ. The claimed dual specificity of Syncytin-1 for two receptors and the explicit involvement of ASCT1 in Syncytin-1-induced fusogenicity have not yet been clarified.

In this study, our objective was to quantitatively compare the individual contribution of ASCT1 and ASCT2 to the interaction of human cells with Syncytin-1 and other RDR viruses. For this purpose, we improved the previously developed heterologous system (40) and abrogated endogenous expression of ASCT1 and ASCT2 in autologous human embryonic kidney (HEK293T) cells. Next, we separately expressed these transporters and evaluated their interaction with Syncytin-1 and other RDR envelopes. Our findings demonstrate that ASCT1 does not contribute to the Syncytin-1-induced cell-to-cell fusion. We address the physiological role of individual receptors in placenta morphogenesis and receptor specificity of RDR viruses.

## Results

### Experimental System to Evaluate ASCT1 and ASCT2 Receptor Function.

To quantify the individual contribution of ASCT1 and ASCT2 to the interaction with the RDR envelope glycoproteins, we developed an in vitro model system based on the HEK293T cell line. Employing CRISPR/Cas9, we sequentially knocked out endogenous ASCT1 and ASCT2 and picked two double knockout clones, 3F9-2E12 (FE) and 3C12-2A9 (CA). Sanger sequencing data suggested that both ASCT1 and ASCT2 were completely knocked out in FE and CA clones. The FE clone revealed homozygous frameshift deletion in the *ASCT1* gene and homozygous single-nucleotide insertion in the *ASCT2* gene (*SI Appendix*, Fig. S1 *A* and *B*). The CA clone contained heterozygous frameshift deletion in *ASCT1* and homozygous frameshift deletion in *ASCT2* (*SI Appendix*, Fig. S1 *A* and *B*). Western blot analysis using anti-ASCT1 and anti-ASCT2 antibodies confirmed the absence of endogenous ASCT1 and ASCT2 protein products in both clones (*SI Appendix*, Fig. S1 *C* and *D*, NC lanes).

**Fig. 1.**
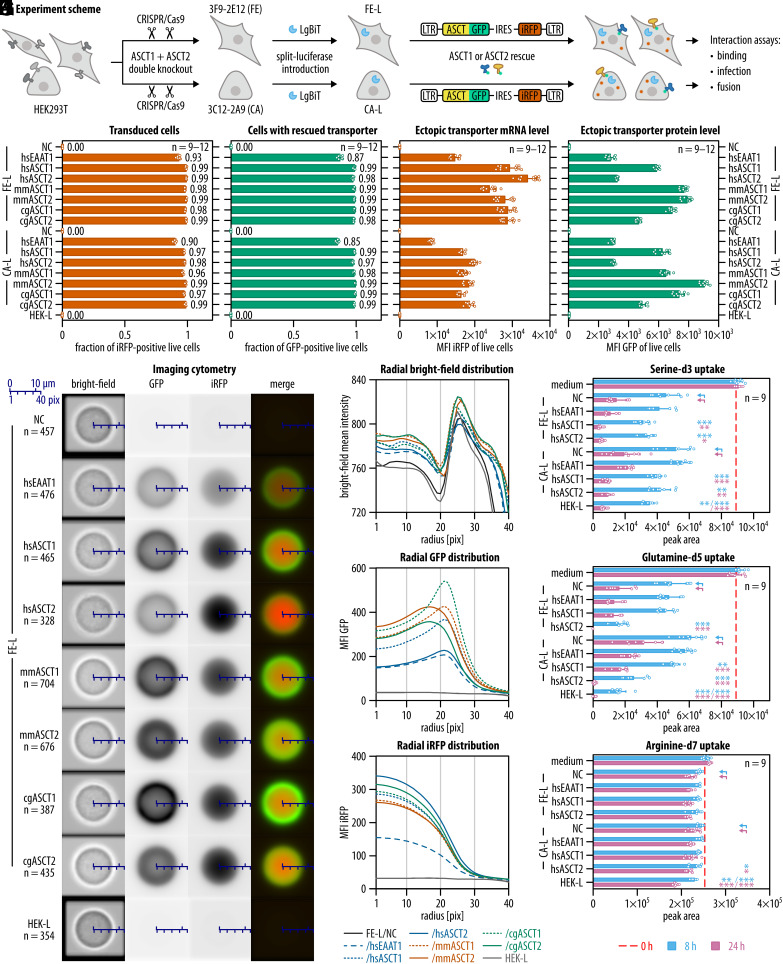
Model system and ectopic expression of investigated transporters. (*A*) Schematic depiction of the experimental system. Using CRISPR/Cas9, we created ASCT1 and ASCT2 double knockout clones. Two double knockout clones, FE and CA (*SI Appendix*, Fig. S1) were used in the experiments. We introduced LgBiT (L) fragment of the NanoLuc luciferase into these clones. Next, we transduced FE-L and CA-L with retroviral constructs encoding GFP-fused EAAT1, ASCT1, or ASCT2 of human (hs), mouse (mm), or Chinese hamster (cg). Transduced, infrared fluorescent protein (iRFP) -positive cells were twice sorted. (*B*) Purity of cells transduced with transporter constructs. (*C*) Fractions of cells expressing ectopic transporters in the living population. (*D*) The levels of transporter construct mRNA quantified as the iRFP mean fluorescence intensities (MFI). (*E*) The levels of transporter protein quantified as MFI of the GFP. (*F*) Localization of ectopically expressed transporters throughout the cells by imaging cytometry. Images of focused cells were aligned and averaged; n represents the number of averaged cells. The averaged images for individual transporters are shown in bright-field, GFP, and iRFP channels, and as composites of the green fluorescent protein (GFP) and iRFP channels (red). The blue scale bars show the radius in pixels and μm. (*G*–*I*) The summarized radial signal distribution from *F* of bright-field (*G*), GFP (*H*), and iRFP (*I*) channels demonstrating cellular localization of transduced proteins. The contrast transition in bright-field points to the cellular edge. (*J*–*L*) Mass spectrometry measurements of deuterated serine (*J*), glutamine (*K*), or arginine (*L*) uptake. The red dashed lines indicate the initial amounts of the labeled amino acids in the medium, and the blue and pink bars show their decrease after 8 or 24 h of cultivation, respectively. All bars are shown as means with SD. Two-way ANOVA with Tukey’s multiple comparison correction was calculated in *J*–*L*, *P* values related to arrow-pointed controls are shown as follows: <0.05: *, <0.01: **, and <0.001: ***. The experiments in *B*–*E* and *J*–*L* were repeated three times and the numbers of total biological replicates are shown as n. In *F*, n corresponds to the number of analyzed and averaged cells. The gating schemes for flow and imaging cytometry are shown in *SI Appendix*, Fig. S2 *A*–*C*. NC, negative control, FE-L or CA-L cells with no ectopic expression.

In our experimental design (Fig. 1*A*), FE and CA clones with abrogated ASCT1 and ASCT2 endogenous expression were employed for either ASCT1 or ASCT2 transporter rescue. The involvement of each transporter in the interaction with Syncytin-1 or other RDR envelopes was further determined using three different quantitative assays described below: i) infectious assay, ii) receptor-binding assay, and iii) fusion assay. The fusion assay was based on the NanoBiT luciferase complementation and required coculturing two pools of cells: one pool expressing the LgBiT (L) fragment of NanoLuc luciferase and the other pool expressing the high-affinity HiBiT (H) fragment of the luciferase (41). To create such pools, we introduced the stable expression of the LgBiT or HiBiT fragments into both double knockout clones as well as the parental HEK293T. The resulting cells are denoted FE-L, FE-H, CA-L, CA-H, HEK-L, and HEK-H.

Next, CA-L and FE-L cells were transduced separately with retroviral constructs encoding either ASCT1 or ASCT2 transporters from human (*Homo sapiens*, hsASCT1, hsASCT2), mouse (*Mus musculus*, mmASCT1, mmASCT2), and Chinese hamster (*Cricetulus griseus*, cgASCT1, cgASCT2) (Fig. 1*A*). As a negative control, we transduced the human Excitatory Amino Acid Transporter (hsEAAT1, SLC1A3), which is structurally similar to ASCT1 and ASCT2, but does not interact with RDR envelopes (40). All transporters were C-terminally fused with GFP, and their mRNA also encoded iRFP, which was cap-independently translated from the same mRNA using internal ribosome entry site (IRES) (Fig. 1*A*). The presence of iRFP allowed for unambiguous separation of all transduced, iRFP-positive, cells by fluorescence-activated sorting, regardless of transporter protein levels. In turn, GFP fused with the transporter permitted straightforward quantification of transporter expression.

After two subsequent sorts, we detected stable ectopic expression of transporter-containing constructs in more than 90% of cells in the populations (Fig. 1*B*) with transporters being expressed in at least 85% of cells (Fig. 1*C* and *SI Appendix*, Fig. S2 *A* and *B*). The mRNA levels, represented by iRFP MFI, differed between FE-L and CA-L cells (Fig. 1*D*), but the differences in transporter protein levels, represented by GFP MFI, were similar in both clones (Fig. 1*E*). The observed protein levels did not fully correspond to the abundance of their mRNA, showing that hsEAAT1, hsASCT2, and cgASCT2 had lower protein levels than hsASCT1, mmASCT1, mmASCT2, and cgASCT1 (Fig. 1*E*). The successful ectopic expression of hsASCT1- and hsASCT2-fusion proteins was also visualized by western blots (*SI Appendix*, Fig. S1 *C*–*F*). In the next step, we verified protein localization by imaging cytometry (Fig. 1*F* and *SI Appendix*, Figs. S2 *C* and *D* and S3). We calculated the average radial signal distribution for bright-field, GFP, and iRFP across the cells. While iRFP was uniformly distributed throughout the cytoplasm, all transporters were displayed mainly on the cell surface (Fig. 1 *G*–*I* and *SI Appendix*, Figs. S2 *E*–*G* and S3) and, therefore, accessible to viral envelope glycoproteins.

Finally, we verified the proper folding of the examined human transporters by measuring the cellular uptake of their substrate. Glutamine was expected to be transported preferentially by ASCT2 (30), while serine should be transported by both ASCT1 and ASCT2 (27, 30). As a nonspecific control, we used arginine because this amino acid is not the substrate for any of the tested transporters. Cells were grown in medium containing deuterated serine, glutamine, and arginine, and their decrease in concentration from the culture supernatants was measured by mass spectrometry after eight and 24 h. Both clones with endogenous ASCT1 and ASCT2 knocked out (NC) showed slower uptake of deuterated amino acids compared to HEK-L (Fig. 1 *J*–*L*). The overexpression of hsEAAT1 did not affect the uptake of the tested amino acids, as it transports glutamate and aspartate. However, transduction of hsASCT1 or hsASCT2 increased the serine cellular uptake (Fig. 1*J*) and, in addition, the glutamine uptake in the case of hsASCT2 (Fig. 1*K*). The minor effect on arginine uptake was presumably not receptor-specific (Fig. 1*L*). The obtained results were consistent with the observed substrate specificity of the transporters, demonstrating that the ectopically expressed proteins were correctly folded to perform their physiological function in the cell.

Taken together, we developed a robust in vitro model system for expression of hsASCT1 and hsASCT2 in human cells. The specific amino acid uptake from the culture medium indicates their proper folding. Fluorescence analysis showed that the human transporters, together with their mouse and Chinese hamster orthologues, were exposed on the cell surface.

### RDR Viruses Considerably Differ in ASCT1/ASCT2 Receptor Usage.

**Fig. 2.**
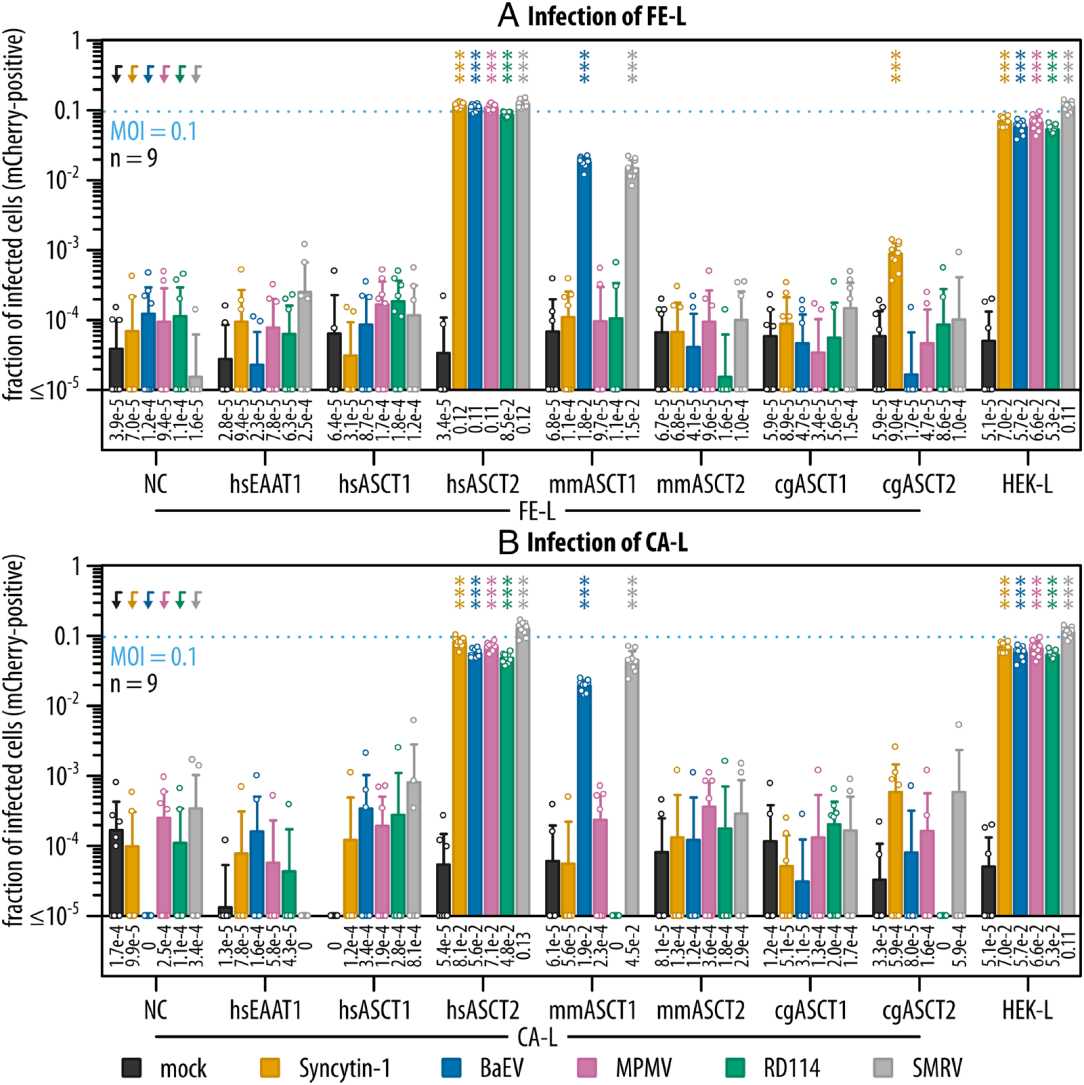
Evaluation of ASCT1 and ASCT2 receptor function for viruses of the RDR interference group. (*A* and *B*) Double knockout clones FE-L and CA-L expressing different transporters were infected with pseudotypes of Syncytin-1, BaEV, MPMV, RD114, or SMRV with MOI 0.1 or mock infected. The pseudotypes transduced mCherry fluorescent marker, the fractions of mCherry-positive cells represent fractions of infected cells. Dotted lines indicate expected infection rates with MOI 0.1. All data are shown as means with SD; the means are also reported as numerical values below the bar plots. Mann–Whitney nonparametric statistical tests with Holm–Šídák multiple comparison correction were performed, *P* values related to arrow-pointed controls are following: <0.001: ***. The data were collected together in three independent experiments and shared the same positive control of HEK-L cells. The total number of biological replicates is shown as n. The gating scheme is shown in *SI Appendix*, Fig. S4*A*. NC, negative control, FE-L or CA-L cells with no ectopic expression.

Next, we used our ASCT1/2 expression system to quantitatively compare the capacity of both transporters to serve as receptors for Syncytin-1 and other members of the RDR interference group. Syncytin-1 and envelopes of BaEV, MPMV, RD114, and SMRV pseudotyped a nonreplicative avian leukosis virus transducing fluorescent marker mCherry. In addition to human ASCT1/2 transporters, we also tested their orthologues from the mouse and Chinese hamster. As a control of infection, we used HEK-L cells that were naturally sensitive to all RDR viruses (Fig. 2 *A* and *B*).

Both double knockout clones expressing different variants of ASCT1 or ASCT2 were infected with the same 0.1 multiplicity of infection (MOI) (Fig. 2 *A* and *B*) and assessed by flow cytometry. The gating strategy for infectivity assays is depicted in *SI Appendix*, Fig. S4*A*. The negative controls, FE-L or CA-L cells without expression of ectopic transporters (NC), displayed a very low background of mCherry-positive cells after infection (Fig. 2 *A* and *B*). We observed that overexpression of hsEAAT1 did not alter the resistant phenotype. On the other hand, expression of hsASCT2 conferred sensitivity to all virus pseudotypes tested. While mmASCT1 conferred sensitivity to BaEV and SMRV pseudotypes, both mmASCT2 and cgASCT1 were completely resistant to all RDR pseudotypes. Surprisingly, cgASCT2 displayed sensitivity to the Syncytin-1 pseudotype above the experimental background. Importantly, we did not detect any infection of cells expressing hsASCT1 under MOI 0.1.

In order to further enhance the possibility of infection, we spinoculated the highest available infectious titer of individual pseudotypes onto the cells (MOI between 0.5 and 6, depending on the particular pseudotype). In this setup, hsASCT1-expressing cells showed low sensitivity only to SMRV and BaEV pseudotypes, but not to Syncytin-1 (*SI Appendix*, Fig. S5). Additionally, we detected successful infection of cells expressing mmASCT1 with all pseudotypes, with the exception of Syncytin-1. Taken together, RDR viruses differ in their receptor usage. While hsASCT2 is a receptor for all RDR viruses, hsASCT1 was much less efficient and, surprisingly, it did not confer sensitivity to Syncytin-1 pseudotype. Moreover, we described the receptor specificity of MPMV and SMRV.

### ASCT1 Is Not a Physiologically Relevant Receptor for Syncytin-1.

**Fig. 3.**
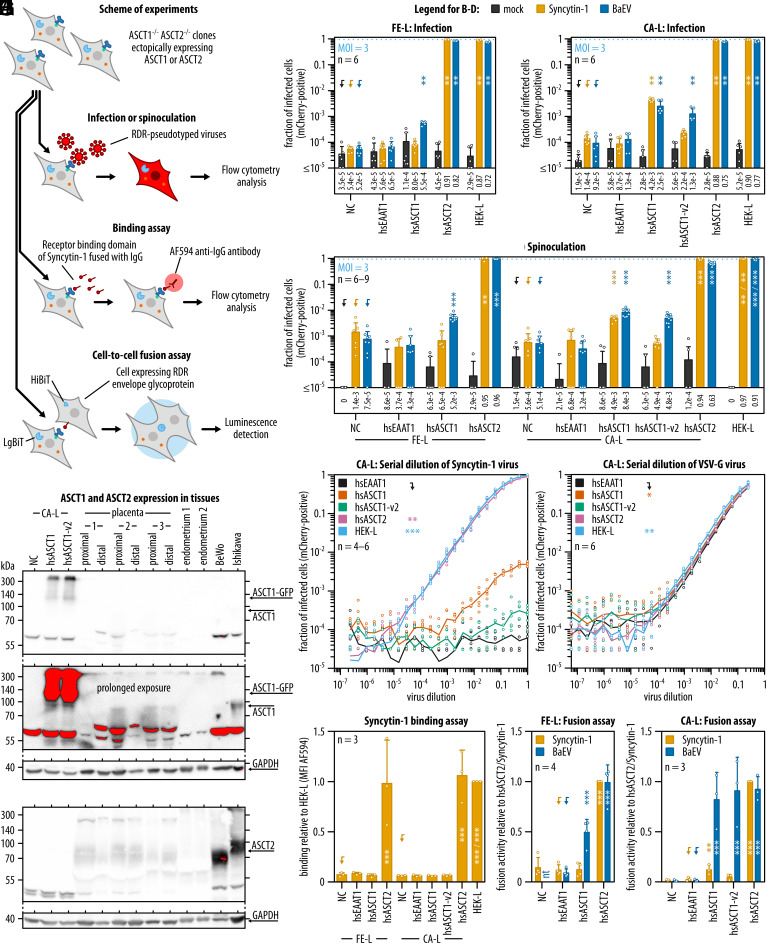
Evaluation of the interaction of ASCT1 with Syncytin-1 and BaEV. (*A*) Schematic depiction of the experimental approach. (*B*–*D*) Double knockout clones FE-L and CA-L expressing different human transporters or HEK-L cells were infected or spinoculated with pseudotypes of Syncytin-1 or BaEV with MOI 3, or mock infected. Dotted lines indicate expected infection rates with MOI 3. The pseudotypes transduced mCherry fluorescent marker; hence, the fractions of mCherry-positive cells represent fractions of infected cells. The means are reported as numerical values below the bar plots. (*E* and *F*) Syncytin-1 and VSV-G pseudotypes were serially diluted and HEK-L or CA-L cells expressing human transporters were infected. The lines represent the mean fraction of infected cells at individual virus dilution. (*G*) Syncytin-1 binding assay. Cells were incubated with medium containing fusion protein of Syncytin-1 receptor-binding domain and rabbit heavy chain of immunoglobulin G (IgG) and stained with secondary antibody conjugated with Alexa Fluor 594 (AF594). The binding of Syncytin-1 to cells was represented by MFI analyzed by flow cytometry. (*H*–*I*) Fusion assay. FE-H or CA-H cells were transfected with Syncytin-1 or BaEV envelope and cocultured with FE-L or CA-L cells expressing LgBiT and one of the tested transporters. The interaction between the envelope and the receptor triggered cell-to-cell fusion, which led to the complementation of NanoLuc luciferase. The signal was integrated and adjusted to the fraction of transfected cells. (*J*) Western blot comparison of ectopic ASCT1 expression in CA-L and CA-L/hsASCT1 cell lines, or endogenous ASCT1 and ASCT2 expression in human distal or proximal placenta sections and human endometrium tissues, BeWo choriocarcinoma cell line and Ishikawa endometrial carcinoma cell line. GAPDH was used as a loading control. For ASCT1, two different expositions are shown. Saturated pixels are highlighted in red. Expected sizes of the proteins are depicted by arrows. All bars are shown as means with SD. Statistical tests: Mann–Whitney with Holm–Šídák multiple comparison correction (*B*–*D*), Kruskal–Wallis test of areas under curves with Dunn's multiple comparison correction (*E* and *F*), one-way ANOVA with Tukey’s multiple comparison correction (*G*), two-way ANOVA with Dunnett’s multiple comparison correction (*H* and *I*). *P* values related to arrow-pointed controls are shown as follows: <0.05: *, <0.01: **, and <0.001: ***. Experiment *H* was repeated four times, other experiments three times. The total numbers of replicates are shown as n. NC, negative control, FE-L or CA-L cells with no ectopic expression; nt, not tested.

The results showing that Syncytin-1 uses only hsASCT2 but not hsASCT1 challenged previously published findings (23), so we focused on this question further. In a pilot experiment, we observed a few mCherry-positive cells after infection of CA-L/hsASCT1 with Syncytin-1-pseudotyped virus. To evaluate the specificity of this phenotype, we recreated CA-L cells with reconstituted expression of hsASCT1 as a second biological duplicate. These cells were denoted CA-L/hsASCT1-v2. We verified that both CA-L/hsASCT1 and CA-L/hsASCT1-v2 expressed hsASCT1 at the same levels, were physiologically active, and were present on the cell surface (*SI Appendix*, Fig. S6 *A*–*K*). To investigate the existence of interaction between hsASCT1 and Syncytin-1, we applied three quantitative assays: i) infection with Syncytin-1 pseudotype, ii) receptor binding of soluble Syncytin-1, and iii) detection of Syncytin-1-induced fusion (Fig. 3*A*).

For the infection measurements, the experiment was scaled up from 96-well to 12-well formats to get a larger ratio of susceptible cells to the experimental background. Further, we used the highest available but equal infectious titers of Syncytin-1 and BaEV pseudotypes to infect the cells expressing human transporters (MOI 3). The BaEV pseudotype infected 0.05 to 0.3% of cells expressing hsASCT1 and more than 75% of cells expressing hsASCT2. The Syncytin-1 pseudotype infected at least 88% of hsASCT2-expressing cells, but, in contrast, only 0.4% of CA-L/hsASCT1 cells, 0.02% of CA-L/hsASCT1-v2, and 0.01% of FE-L/hsASCT1 (Fig. 3 *B* and *C* and *SI Appendix*, Fig. S4*B*). The background on CA-L/NC and FE-L/NC was 0.01%. We also spinoculated both pseudotypes in a 96-well format with MOI 3. We observed a three- to ninefold increase in hsASCT1 infection with the BaEV pseudotype, whereas there was essentially no increase with the Syncytin-1 pseudotype (Fig. 3*D*). The background level increased up to 0.1% of mCherry-positive cells.

To assess the interaction dynamics between Syncytin-1 and hsASCT2 vs. hsASCT1 in CA-L cells, we quantified the infection with serially diluted pseudotypes of Syncytin-1 or VSV-G (Fig. 3 *E* and *F*). CA-L/hsASCT2 showed the same sensitivity as HEK-L (Fig. 3*E*). We observed a 100 to 1,000× difference in sensitivity to Syncytin-1 pseudotype between CA-L/hsASCT2 and CA-L/hsASCT1. CA-L/hsASCT1-v2 infection remained at the background level and was not statistically different from CA-L/hsEAAT1 infection. All cells showed comparable sensitivity to the VSV-G pseudotype, which uses an unrelated broadly expressed receptor (42) (Fig. 3*F*).

Next, we evaluated the binding of Syncytin-1 in the form of soluble immunoadhesin to the individual hsASCT1/2 transporters (Fig. 3*G*). Immunoadhesin represents the fusion protein of the Syncytin-1 receptor-binding domain and the heavy chain of IgG. The receptor-binding assay involved incubating living cells with the immunoadhesin and quantification of receptor-bound Syncytin-1 using a fluorescently labeled anti-IgG antibody (40). Dilution of the immunoadhesin proved concentration-dependent binding to the hsASCT2 receptor but not to the hsEAAT1 transporter (*SI Appendix*, Fig. S7*A*). Moreover, the specificity of immunoadhesin toward ASCT2 was confirmed by immunoprecipitation, followed by mass spectrometry analysis of precipitated proteins. The data demonstrated that immunoadhesin is bound to the ASCT2 protein while ASCT1 was not detected (*SI Appendix*, Fig. S7*B*). Using our model cell lines as a substrate for the binding assay, we observed that the soluble Syncytin-1 bound to hsASCT2-expressing cells to the same extent as to HEK-L cells, whereas the binding to hsASCT1 remained the same as that of hsEAAT1 or NC (Fig. 3*G*).

Finally, we compared the capacity of both transporters to induce cell-to-cell fusion (Fig. 3 *H* and *I*). The fusion assay was based on the cocultivation of cells overexpressing the envelope glycoproteins and a HiBiT with cells expressing the transporter and the other part of split luciferase (LgBiT). The interaction of the fusogenic envelope glycoprotein with the specific receptor triggered fusion of cells, which led to luciferase complementation, and light emission. We observed the formation of multinucleated syncytia (*SI Appendix*, Fig. S8) and detected robust luciferase signal after hsASCT2 interaction with Syncytin-1 or BaEV envelope glycoprotein. In comparison to hsASCT2, we observed 49% to 91% of fusion triggered by the interaction between BaEV envelope glycoprotein and hsASCT1. These results correspond well with the BaEV infectivity results (Fig. 3 *B* and *C*) and confirm hsASCT1 receptor function for BaEV. In contrast, Syncytin-1 fusogenic activity following the interaction with hsASCT1 reached maximally 12% in the CA-L clone and remained at background levels in the FE-L clone.

To evaluate the relevance of our findings for in vivo cell-to-cell fusion (*SI Appendix*, Fig. S9*A*), we examined ASCT1 expression levels in human placenta and endometrial tissues and compared them to the BeWo choriocarcinoma cell line, the Ishikawa epithelial endometrial carcinoma cell line, and our model cell lines. As a positive control of expression in human tissues and cells, we used ASCT2. We easily detected ASCT2 in human placental tissues and in BeWo and Ishikawa cells, but not in the endometrial tissues (Fig. 3*J*). ASCT1, the primary protein of interest in this experiment, was not detected in endometrial tissues but it was identified in low amounts in Ishikawa cells and in healthy term placentas (Fig. 3*J*). The ASCT1 level in term placentas was detectable on the side facing maternal decidua, although it was considerably lower than the hsASCT1-GFP fusion protein level in our CA-L/hsASCT1 cell lines. Also, the single-cell RNA sequencing data from the first-trimester maternal–fetal interface (43) showed low levels of *ASCT1* mRNA (*SI Appendix*, Fig. S9*B*). These results support our findings that ASCT1 is not involved in the cell-to-cell fusion in the human placenta.

These results demonstrated that Syncytin-1 interacts primarily with human ASCT2. The interaction with human ASCT1 was close to the background and appeared only under specific conditions of high abundance of Syncytin-1. Considering the high hsASCT1 expression in our model system, we conclude that ASCT1 does not participate in Syncytin-1-mediated fusion.

### Chinese Hamster ASCT2 Forms Heterotrimers with Human ASCT1 and Can Complement the Function of Syncytin-1 Receptor.

**Fig. 4.**
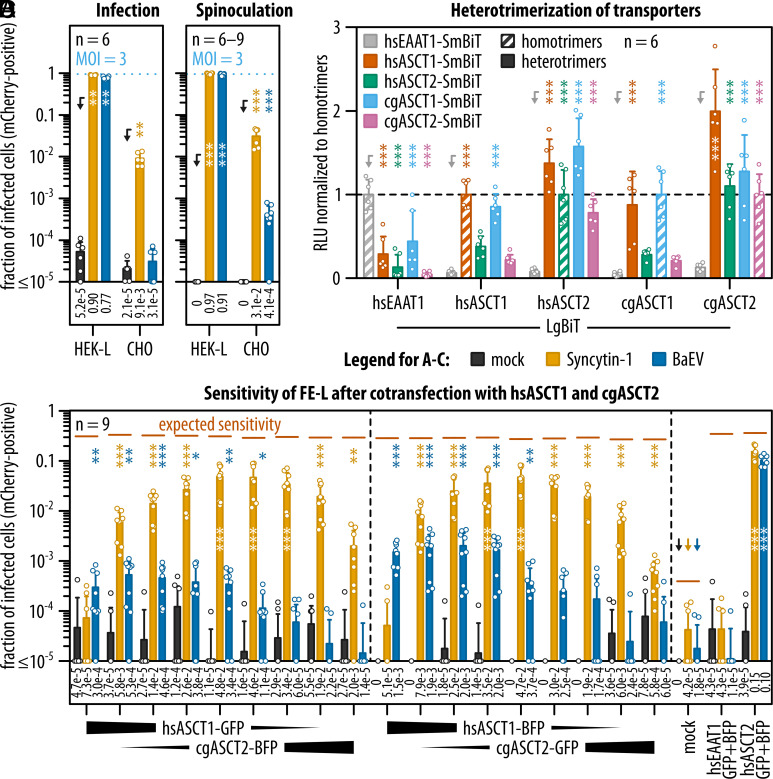
Sensitivity of Chinese hamster receptors to Syncytin-1 and BaEV pseudotypes and receptor heterotrimers evaluation. (*A* and *B*) Flow cytometry analysis of HEK-L and CHO cells after infection or spinoculation with Syncytin-1 or BaEV pseudotypes. Data are shown as means with SD; the means are also reported as numerical values below the bar plots. Dotted lines indicate expected infection rates with MOI 3. (*C*) Sensitivity to infection of FE-L cells coexpressing human ASCT1 and Chinese hamster ASCT2 fused with either GFP or blue fluorescent protein (BFP). The ratios of transiently transfected plasmids were 100:0, 99:1, 96:4, 90:10, 50:50, 10:90, 4:96, 1:99, and 0:100. 2 d after the transfection, the cells were infected with MOI 3 or mock infected and the infection was quantified by flow cytometry. Data are shown as means with SD; the means are also reported as numerical values below the bar plots. The gating strategy is shown in *SI Appendix*, Fig. S11*D*. Expected rates of infection according to fraction of transfected cells (*SI Appendix*, Fig. S12) and used MOI are depicted by dashes. (*D*) Luminescence quantitative assay determining the colocalization of transporters fused with the LgBiT or SmBiT parts of NanoLuc luciferase in FE cells. The relative luciferase units (RLU) were normalized to respective homotrimers shown as hatched bars. The bars represent average values with SD. Statistical tests: Mann–Whitney with Holm–Šídák multiple comparison correction (*A*–*C*), two-way ANOVA with Dunnett’s multiple comparison correction (*D*). *P* values related to arrow-pointed controls are shown as follows: <0.05: *, <0.01: **, and <0.001: ***. All experiments were repeated three times; the total numbers of biological replicates are shown as n.

The central finding of our study, the receptor insufficiency of human ASCT1 for Syncytin-1-pseudotyped virus, is in striking contrast to Lavillette et al. (23), who observed that Chinese hamster ovary (CHO) cells turned sensitive to Syncytin-1 after transient transfection with hsASCT1. In our infectivity experiments, we have seen a weak interaction of Syncytin-1 with cgASCT2 ectopically expressed in FE-L double knockout cells (Fig. 2*A*). We next asked whether the level of endogenous ASCT2 expression is sufficient for CHO cells infection with Syncytin-1 pseudotypes transducing mCherry. In correspondence to the weak Syncytin-1–cgASCT2 interaction, we detected 0.9% of CHO cells infected with the Syncytin-1 pseudotype at MOI 3 (Fig. 4*A*) and 3% after spinoculation (Fig. 4*B*). It is worth noting that the sensitivity of CHO cells to Syncytin-1 was even higher than that of all tested hsASCT1-expressing cells (Fig. 3 *B* and *C*). This observation corresponds with the weak receptor activity of cgASCT2 for Syncytin-1; nevertheless, it does not explain the results of Lavillette et al. (23), who found that transient transfection of hsASCT1 was comparable to hsASCT2 in providing CHO cells with sensitivity to Syncytin-1. We hypothesized that the increase in sensitivity may be caused by heterotrimerization of endogenous, sensitive ASCT2 with abundant, transfected hsASCT1.

At this point, we investigated the susceptibility of cells coexpressing cgASCT2 with other similar transporters. We transiently cotransfected FE-L cells with a 1:1 ratio of human and hamster transporters fused with GFP or BFP in reciprocal combinations. We analyzed the expression of the respective transporters using flow cytometry (*SI Appendix*, Fig. S10). A substantial fraction of transfected iRFP-positive cells expressed both receptors, with percentages ranging from 42% to 72% (*SI Appendix*, Fig. S10*E*). Simultaneously with this flow cytometry analysis, we infected the transfected cells with viruses pseudotyped with Syncytin-1 or BaEV at MOI 3. As expected, the highest infection rate by each virus was detected in cells expressing hsASCT2, regardless of the fluorescent tag or cotransfected transporter (*SI Appendix*, Fig. S11). In contrast, hsASCT1-only transfected cells remained resistant to Syncytin-1 (*SI Appendix*, Fig. S11*B*). We confirmed the weak specificity of Syncytin-1 for cgASCT2, and, in accordance with our hypothesis, cgASCT2 combined with hsASCT1 conferred a slightly lower sensitivity than hsASCT2 alone. Four percent of cells coexpressing the reciprocal combinations of cgASCT2-hsASCT1 were sensitive to infection with the Syncytin-1 pseudotype. In comparison, 11% of the hsASCT2-expressing cells were susceptible to infection (*SI Appendix*, Fig. S11*B*). hsASCT1-expressing cells were slightly more infected with BaEV than with the Syncytin-1 pseudotype. However, we did not observe any increase in the sensitivity to BaEV in the case of hsASCT1-cgASCT2 combination (*SI Appendix*, Fig. S11*C*). This indicates that the receptor recognition of Syncytin-1 and BaEV differs.

Next, we examined the complementation of cgASCT2 with hsASCT1 and transfected the FE-L cells with their expression constructs in different ratios (Fig. 4*C* and *SI Appendix*, Fig. S12). A mere 1% of cgASCT2 was sufficient to increase the sensitivity to Syncytin-1 by two orders of magnitude when hsASCT1 was in excess. The cellular sensitivity peaked at equimolar ratio of cgASCT2 to hsASCT1 (Fig. 4*C*). In contrast, BaEV infection corresponded only to hsASCT1 amount. This result demonstrated that hsASCT1 and cgASCT2 complement each other and boost cellular sensitivity to Syncytin-1.

Finally, to test whether the molecular mechanism of complementation between cgASCT2 and hsASCT1 is formation of heterotrimers, we fused both transporters with LgBiT or SmBiT, the two complementary parts of NanoLuc luciferase with low affinity for each other. After cotransfection of the unmodified FE clone with the LgBiT- and SmBiT-tagged transporters, we measured luciferase complementation caused by close proximity of the tagged proteins (Fig. 4*D* and *SI Appendix*, Fig. S13). We used the luminescence signal obtained from the respective homotrimers as a reference. Formation of heterotrimers between hsEAAT1 and any of ASCTs transporters was ineffective. On the other hand, these experiments showed formation of heterotrimers among ASCT1 and ASCT2 of diverse origins.

In conclusion, our results show that human ASCT1 is not interacting with Syncytin-1. We demonstrated that artificial coexpression of hsASCT1 potentiates the cgASCT2 interaction with Syncytin-1 and suggested that heterotrimerization could be the respective molecular mechanism.

## Discussion

Due to its fusogenic activity, Syncytin-1 is one of the key players in human placenta differentiation. Its ability to induce cell-to-cell fusion was suggested to be mediated by two structurally similar receptors, ASCT2 and ASCT1 (22, 23). This dual specificity of Syncytin-1 has been widely accepted, although it was based solely on receptor overexpression in a heterologous system of CHO cells. The specific contribution of each receptor to Syncytin-1 fusogenic activity has not been investigated. In this study, we sought to fill this gap and quantitatively compare the function of ASCT1 and ASCT2 receptors. Apparently, there is no direct experimental way to assess the involvement of individual receptors in the Syncytin-1-induced fusion process in human placenta tissue. Thus, we used contemporary techniques of gene knockout and gene expression control in the human HEK293T cell line. In a series of experiments, we found that ASCT2 is responsible for most of the interactions of Syncytin-1 with human cells, while ASCT1 is not involved in these interactions.

Our experiments have shown that the efficiency of the ASCT1–Syncytin-1 interaction is reduced by several orders of magnitude in comparison to ASCT2. Considering the low levels of ASCT1 that can be detected at the human maternal–fetal interface (Fig. 3*J* and *SI Appendix*, Fig. S9), the involvement of ASCT1 is even more unlikely. Nevertheless, in an in vivo context, we cannot exclude the possibility that ASCT1 expression may be locally increased, along with the potential impact of posttranslational modifications, e.g., glycosylations. These in vivo conditions could be theoretically sufficient for cell-to-cell fusion. Although the presented experimental cellular model does not mimic the in vivo situation, it effectively considers multiple parameters, including expression level and localization. Moreover, it allows for the quantification of the interaction between Syncytin-1 and its receptor. Collectively, our results demonstrate that ASCT1 does not contribute to the fusogenic activity of Syncytin-1.

To support our conclusions, we modeled the original situation in CHO cells and analyzed the interaction between Syncytin-1 and both cgASCT2 and hsASCT1. Our experiments showed that previous claims of human ASCT1 as a receptor for Syncytin-1 were based on the weak affinity of Syncytin-1 for endogenous Chinese hamster ASCT2 in CHO cells. Furthermore, we demonstrated that Chinese hamster ASCT2 interaction with Syncytin-1 can be enhanced by heterotrimerization with ectopically expressed human ASCT1.

The data presented here were obtained using a model system that upgrades a previously validated system relying on hsASCT2 expression in chicken cells (40). First, to eliminate the experimental background, the innovative design employed CRISPR/Cas9 knockout of endogenous *ASCT1* and *ASCT2* genes in the HEK293T cell line (Fig. 1*A*). Second, our system permitted stable rescue of transporters by feline leukemia virus-based vectors (44). Similar to the previously approved avian system, the C-terminal fusion with GFP enabled tracking of expression and localization of transporters. The efficient display of transporters on the cell surface contributed to high sensitivity of the Syncytin-1 binding and fusion assays (Fig. 3 *G*–*I*). For infection assays, we used pseudotyped nonreplicative alpharetroviral vectors with codon-optimized *gag* and *pol* sequences. The viral particles pseudotyped with Syncytin-1, or envelope glycoproteins of BaEV, MPMV, RD114, or SMRV were produced on the FE double knockout clone, which did not form syncytia after the fusogenic envelope expression (*SI Appendix*, Fig. S8). Together with high efficiency of transfection and transfected gene expression, it resulted in significantly increased viral titers, reaching up to 2 × 10^5^ IU/mL for Syncytin-1 and 6 × 10^5^ IU/mL for BaEV pseudotypes.

Using this experimental model, we have unequivocally demonstrated that human ASCT2 was both necessary and sufficient for Syncytin-1 binding (Fig. 3*G*), cellular entry (Figs. 2 and 3 *B*–*E*), and induction of cell-to-cell fusion (Fig. 3 *H* and *I*). In contrast, the ectopic expression of hsASCT1 did not rescue the human ASCT1/ASCT2 ablation (Figs. 2 and 3), which disqualified human ASCT1 as a bona fide receptor for Syncytin-1. The discrepancy with the previous study (23) stems from the use of CHO cells, which are generally accepted as resistant to RDR viruses (22, 45). Our results surprisingly showed that the Syncytin-1 pseudotype could infect wild-type CHO cells, albeit to a much lesser extent than human cells (Fig. 4 *A* and *B*). In line with this unexpected finding, we demonstrated that the cgASCT2 mediated entry of the Syncytin-1 pseudotype (Fig. 2*A*). This disagreement with previous publications could be explained by higher titer of virus pseudotype. We infected CHO cells with MOI 3, which was not possible in Lavillette et al. (23), who produced the lentiviral pseudotypes at the titer of 1.2 × 10^3^ to 5.2 × 10^4^ CFU/mL on syncytia-forming HEK293T cells. In the Lavillette et al. (23) study, the receptor function of the weakly expressed endogenous ASCT2 in CHO cells (*SI Appendix*, Fig. S1) could have been potentiated by its multimerization with overexpressed hsASCT1. Accordingly, we observed in our experiments at least 20 times higher sensitivity to infection with Syncytin-1 pseudotype after supplementation of cgASCT2 with hsASCT1 (Fig. 4*C* and *SI Appendix*, Fig. S11*B*), which almost reached the sensitivity of cells expressing hsASCT2 alone.

We examined the heterotrimerization of cgASCT2 and hsASCT1 as a molecular mechanism of interaction potentiation. The split-luciferase subunits used, the LgBiT and SmBiT, were optimized for minimal self-association due to their weak affinity (K_d_ = 190 µM) (41); therefore, the complementation of NanoLuc luciferase reflected the proximity of protomers. The luminescence signal of all ASCT-only combinations was higher than that of combinations involving hsEAAT1 (Fig. 4*D*). We assume that the luminescence signal originated specifically from the presence of protomers in the same complex assembled as a heterotrimer. We further checked the signal localization using luminescence microscopy and observed membrane localization in cells expressing the combination of cgASCT2 and hsASCT1 (*SI Appendix*, Fig. S13). Thus, the heterotrimers were accessible to viral envelope glycoproteins. The protomers may transport their substrate independently, as was demonstrated with EAATs (46), and the heterotrimers could be physiologically functional transporters. In contrast to the transporting function, the interacting interface of Syncytin-1 probably uses more protomers (47). The enhancement of the interaction between Syncytin-1 and cgASCT2 by the presence of hsASCT1 could be caused by complementation of the binding site, favoring that more protomers create a single Syncytin-1 binding site, and/or simply by increasing the receptor display on the plasma membrane, suggesting that the binding site is restricted to only one protomer.

Of note, the interaction between hamster and human proteins may occur only under experimental conditions and is not biologically relevant for any species. Even the data presented here on mouse and Chinese hamster receptors have limitations, as the proteins were expressed in a heterologous host. The peaks of the localization signals of mmASCT2 and cgASCT2 were not as sharp as those of human transporters (Fig. 1*H*). This suggests that their expression in human cells was probably suboptimal, and part of the proteins was retained in the secretory pathway (*SI Appendix*, Fig. S3). We do not consider this to be a natural expression pattern, but rather a result of their heterologous expression. Therefore, the sensitivity of these two receptors may be underestimated.

Our experimental system combining the human ASCT1/ASCT2 double knockout, ectopic expression of species-specific ASCT1 or ASCT2, and infection with pseudotyped vectors enabled comprehensive comparison of RDR group receptor usage. Whereas the receptor specificity of Syncytin-1, BaEV, and RD114 has already been thoroughly examined (23, 36, 48, 49), no such experiments were performed with MPMV and SMRV. Primarily, we found that human ASCT2 is the major receptor for all tested RDR members (Fig. 2). Our results are in accordance with the concept of the single RDR interference group (37) and confirm human ASCT1 as an auxiliary receptor for BaEV (36). Notably, mouse ASCT2 turned out to be resistant to all tested viruses, probably due to the insertion of several amino acids into the C-terminal part of ECL2 (48). BaEV, SMRV, and, after spinoculation, RD114 could extend their tropism by using mouse ASCT1. The pattern of SMRV-recognized receptors was the same as that of BaEV and included human ASCT2, mouse ASCT1, and, after spinoculation, human ASCT1. MPMV, on the other hand, interacted only with the human ASCT2. These findings demonstrate the differences in receptor usage within the same interference group and highlight the possibility of cross-species transmissions.

We point out that the results of experiments in heterologous hosts should be interpreted with caution, particularly when dealing with multimeric proteins. Previous virological studies employed unmodified hamster (34, 36, 48, 50–52), mouse (35, 53, 54), rat (22), chicken (40), or human cells (55, 56) for the expression of RDR receptors from other species. Despite well-controlled experiments, potential complementation with endogenous proteins was overlooked. This concern extends beyond ASCT1/ASCT2 to other multimeric retroviral receptors. PiT1 (SLC20A1) or PiT2 (SLC20A2), receptors for another large interference group, including gibbon ape leukemia virus, feline leukemia viruses B and T, and some strains of murine leukemia virus, naturally occur in heterodimeric form (57). Additionally, the PiT1 and PiT2 receptors are influenced by other host-specific factors, such as the FeLIX protein, which is necessary for the interaction of FeLV-T with PiT1 (58, 59), or an unknown hamster protein factor, which inhibits virus infection via the PiT1 or PiT2 receptors (60). Analogically, human cells encode Suppressyn, which regulates the interaction of human ASCT2 with Syncytin-1 (61, 62). All the proteins mentioned above are thought to be parts of the envelopes of endogenous retroviruses. The complexity of interactions forces us to use the most natural settings for protein studies, and we encourage other researchers to do so as much as possible. We emphasize the importance of selecting an appropriate model system and taking advantage of currently available genome editing techniques.

## Materials and Methods

### Ethical Approval and Sample Collection.

The study was approved by the Medical Ethics Committee of the Eberhard Karls University of Tübingen. All samples were obtained with written informed consent and in accordance with the Declaration of Helsinki (2000) guidelines. The samples were deidentified prior to the use in the study. Human tissue samples were collected at the Women’s University Hospital Tübingen, Germany.

The placentas collected were from planned term c-sections of healthy pregnancies (38 to 40 wk) and processed within 1 h. Endometrial samples were taken from women undergoing hysteroscopy for benign reasons and snap-frozen until processing. No maternal or fetal infections were recorded. In brief, the proximal and distal placental sections and endometrial tissues were washed in phosphate-buffered saline (PBS, Sigma-Aldrich, Catalog #D8537) to remove any blood or connective tissue/contaminants. Subsequently, the tissues were homogenized in ice-cold Radioimmunoprecipitation Assay (RIPA) buffer (ThermoFisher, Catalog #89901), supplemented with freshly added protease inhibitors (Roche, Catalog #05892970001) and homogenized using a sterile hand pestle. Homogenization was carried out on ice until a uniform lysate was obtained. The homogenized samples were then centrifuged at 13,000×*g* for 10 min at 4 °C to pellet cellular debris and other insoluble components. The resulting supernatant containing solubilized proteins was carefully collected and transferred to fresh tubes for further analysis.

### Cloning of Expression Vectors and Viruses.

The design of expression vectors was based on the previously published FuTraP system (40). The coding sequences of hsEAAT1 (GenBank Accession No. NP_004163.3) and hsASCT2 (NP_005619.1) were amplified from BeWo choriocarcinoma cell line cDNA. mmASCT1 (AAG02179.1) and mmASCT2 (NP_033227.2) coding sequences were acquired from plasmids kindly provided by Takayuki Miyazawa, Kyoto University. cgASCT1 (NP_001233711.1) and cgASCT2 (XP_027287146.1) were amplified from CHO-K1 cell line cDNA. hsASCT1 (NP_003029.2) was commercially synthesized (Integrated DNA Technologies) with the GC-rich regions restored by annealing of shorter oligonucleotides. The transporters were cloned into expression plasmids containing the following features: feline leukemia virus long terminal repeat (FeLV LTR, NC_001940.1) promoter, murine leukemia virus (MLV, NC_001502.1) packaging signal, Kozak sequence, coding sequence of transporter fused via 3× G4S linker with superfolder GFP (FPbase ID: B4SOW), encephalomyocarditis virus internal ribosomal entry site (IRES, MG550106.1), iRFP coding sequence (FPbase ID: DGXFA), FeLV LTR, high-copy number ori with mutation in RNAII promoter (63), and ampicillin resistance. Variants with GFP replaced with BFP (FPbase ID: ZO7NN), LgBiT fragment of NanoLuc luciferase (LgBiT, Promega), or SmBiT fragment of NanoLuc luciferase (SmBiT, Promega) were prepared. For transductions of the vectors, we employed pVSV-G plasmid encoding the vesicular stomatitis virus glycoprotein (VSV-G) and MLV Gag and Pol (64) (provided by Axel Schambach, Institute of Experimental Hematology, Hannover).

The coding sequence of Syncytin-1 (NP_001124397.1) was derived from previously used pMCAS(Sync1-MSC16)dsRed (40). BaEV envelope glycoprotein (YP_009109691.1), SMRV envelope glycoprotein (NP_041262.1), and RD114 envelope glycoprotein (YP_001497149.1) were synthesized commercially (Integrated DNA Technologies). MPMV envelope glycoprotein (NP_056894.1) was subcloned from pSARM4 kindly offered by Michaela Rumlová, University of Chemistry and Technology, Prague. All envelope glycoproteins had truncated cytoplasmic domain, as was described previously for Syncytin-1 MSC16 (23, 40), BaEV R-less (23), RD114 R-less (23), and analogically for SMRV (stop-codon after glycine 555) and MPMV (stop-codon after valine 586). Envelope glycoprotein open reading frames were cloned into pVSV-G downstream of the cytomegalovirus (CMV) promoter and the beta-globin intron sequence by replacing the VSV-G open reading frame. Propagation of pseudotypes was ensured by codon-optimized alpharetroviral *gag* and *pol* (65). As the transduced marker genome, we used pAlpha-SF-mCherry-wPRE derived from pAlpha-SF-EGFP-wPRE (65) by exchange of the gene encoding fluorescent protein (mCherry, FPbase ID: ZERB6).

For the majority of cloning steps, an In-Fusion Cloning Kit (TaKaRa) was used. To reduce the risk of recombination, the plasmids were produced in the NEB Stable strain of Escherichia coli in 30 °C. Whole coding sequences were verified by Sanger sequencing.

### Cell Lines, Transfections, and Transductions.

HEK293T cells were grown in DMEM high glucose medium (Sigma) supplemented with 5% of calf serum and 5% of fetal calf serum (DMEM55). CHO-K1 cells were grown in DMEM:F12 media mixture (Sigma) supplemented with 5% of calf serum and 5% of fetal calf serum (NP55). All cells were grown in 37 °C, 5% CO_2_ and humidified atmosphere with addition of 100 units of penicillin and 100 µg of streptomycin per milliliter of media. For all transfections, Lipofectamine 3000 (ThermoFisher) was used according to the manufacturer’s instructions on cells in the exponential phase of growth.

The generation of ASCT1 and ASCT2 double knockout clones FE and CA is described in *SI Appendix*, Fig. S1. Both clones, as well as the parental HEK293T, were transfected with either the CMV HaloTag®-LgBiT or CMV HaloTag®-HiBiT vector (Promega) and selected for 2 wk with 200 µg/mL of Hygromycin B (Roche). The resulting cells expressing LgBiT are denoted as FE-L, CA-L, and HEK-L, respectively. Similarly, cells expressing HiBiT are denoted as FE-H and CA-H.

FE-L and CA-L cells were modified by transduction to ectopically express EAAT1, ASCT1, or ASCT2 from the human, mouse, or Chinese hamster. Each of the transducing viruses were prepared by cotransfection of FE cells by MLV gagpol, pVSV-G, and the plasmid encoding one of the transporters in the ratio 3:2:5. The medium containing transducing viruses was collected 3 d posttransfection, filtered through a 0.45 µm filter and supplemented with polybrene (4 µg per mL). A total of 1.5 × 10^5^ FE-L or CA-L cells on a 6-well plate were infected with 2 mL of the transducing viruses. The cells were expanded to P100 dish and one week after the infection, the population of iRFP-positive cells was sorted by fluorescence-activated cell sorting (FACS). After another 2 wk, the sorting was repeated resulting in populations characterized in Fig. 1. The CA-L/hsASCT1-v2 cells were prepared independently by the same approach.

### Flow and Imaging Cytometry.

The cells were characterized by FACSymphony (BD) flow cytometer or by Amnis ImageStreamX Mk II (Cytek) imaging flow cytometer. The sample preparation with gating strategy is described in *SI Appendix*, Fig. S2.

### Amino Acid Uptake.

All tested cell lines were seeded in biological triplicates on a 96-well plate at 4 × 10^4^ cells/well. After 24 h of incubation, cells were gently washed with PBS, and the cultivation medium was exchanged for DMEM:F12 media mixture without glutamine (Sigma). Cells were cultivated for 24 h, and the medium was exchanged for 140 µL of DMEM:F12 media mixture without glutamine (Sigma) supplemented with deuterated amino acids L-Glutamine-2,3,3,4,4-d5, L-Serine-2,3,3-d3, and L-Arginine-2,3,3,4,4,5,5-d7 (CDN Isotopes), at a final concentration of 100 µM for each. As a negative control, a medium with deuterated amino acids was used in cell-free samples. A volume of 120 µL of supernatant, including the negative control, was collected from all samples after 8 h and 24 h of incubation at 37 °C, 5% CO_2_, and humidified atmosphere. Collected samples were immediately centrifuged at 300×*g* for 5 min at 4 °C. Supernatants were then transferred per 100 µL to new microtubes, and samples were frozen at −80 °C before the subsequent analysis by mass spectrometry. The analysis is described in detail in the Extended materials and methods.

### Western Blotting.

Fifty micrograms of cells or tissues was lysed in a RIPA Lysis and Extraction Buffer (ThermoFisher Scientific), mixed with an SDS-containing sample buffer, subjected to sodium dodecyl sulfate–polyacrylamide gel electrophoresis, and transferred onto polyvinylidene fluoride membrane. ASCT1 was detected using a rabbit polyclonal anti-ASCT1 antibody (#8442, Cell Signaling Technology) diluted 1:1,000. ASCT2 was detected using a rabbit polyclonal anti-ASCT2 antibody (Sigma-Aldrich, HPA035240) diluted 1:700. Horseradish peroxidase–conjugated secondary goat anti-rabbit antibody (Cell Signaling Technology) and SuperSignal West Pico PLUS (Thermo Scientific) were used for chemiluminescence detection. Protein bands were visualized by using iBright™ CL1000 Imaging System (Invitrogen). GAPDH loading control was detected by rabbit monoclonal antibody (#2118, Cell Signaling Technology) diluted 1:1,000 and visualized as above-mentioned.

### Infections.

The viruses pseudotyped with Syncytin-1, BaEV, MPMV, RD114, or SMRV envelope glycoproteins were prepared by transfection of FE cells with alpharetroviral gagpol, pAlpha-SF-mCherry-wPRE, and plasmids encoding envelope glycoprotein in ratio 7:7:1. The pseudotypes were produced to NP55 medium and reached titers of 2 × 10^5^, 6 × 10^5^, 4 × 10^5^, 3 × 10^5^, and 5 × 10^4^ IU/mL, respectively. 1 d before infection, cells were seeded on 96-well (1 × 10^4^, Fig. 2) or 12-well plates (1 × 10^5^, Figs. 3 *B* and *C* and 4*A* and *SI Appendix*, Fig. S4). Prior to infection, viruses were diluted with NP55 to a final volume of 0.1 or 1 mL per well, respectively. The next day, the medium was exchanged for DMEM55. 3 d postinfection, cells were analyzed by flow cytometry. Infected cells were positive for mCherry. The sample preparation with gating scheme is described in *SI Appendix*, Fig. S4*A*. Spinoculations (Figs. 3*D* and 4*B* and *SI Appendix*, Fig. S5) were performed on 96-well plates. After addition of virus, the plates were centrifuged at 1,200×*g*, 23 °C for 2 h. Then, the medium was exchanged, and cells were analyzed by flow cytometry 3 d postinfection. All infections were done in the BSL2 containment. The expected infection rates are characterized by Poisson distribution and are equal to 1 – e^–MOI^.

### Syncytin-1 Binding Assay.

The soluble form of Syncytin-1 fused with the Fc part of IgG gene, so-called immunoadhesin, was used for receptor binding assay as reported recently (40). The immunoadhesin fusion protein consisted of the following domains: the original Syncytin-1 signal peptide, Syncytin-1 amino acids 23 to 152 containing the putative receptor-binding domain, Tobacco Etch Virus protease recognition sequence as a linker, amino acids 175 to 402 of the constant region of the rabbit IgG gene (GenBank Accession No. K00752.1), G4S linker, and the Twin-Strep-tag. The Fc part of the rabbit IgG gene was modified to produce the monomeric protein by insertion of five substitution mutations (66). The immunoadhesin fusion protein was inserted into an expression plasmid downstream of a CMV promoter and the plasmid was transfected into the FE-L cells. 3 d posttransfection, the medium with soluble immunoadhesin was harvested, filtered through a 0.45 μm filter, and stored at −80 °C for further use in the receptor binding assay as previously described (40). Briefly, the cells expressing different transporters, or the negative control FE-L or CA-L cells were detached using a nonenzymatic cell dissociation solution and incubated with the medium containing immunoadhesin at 4 °C for 1 h. Then, the cells were incubated for 30 min at 4 °C with anti-rabbit IgG conjugated to Alexa Fluor 594 antibody (1:1,000 dilution in PBS with 2% bovine serum). After staining, the median fluorescence intensity of Alexa Fluor 594 (ex. 561 nm, em. 610/20 nm) was determined by flow cytometry.

### Cell-to-Cell Fusion Quantification Based on the NanoBiT Split-Luciferase System.

To quantify cell-to-cell fusion initiated by Syncytin-1, we used a similar approach that we have already described in our previous work (40). Our system utilizes high-affinity fragments of NanoLuc luciferase – LgBiT and HiBiT, which together form an enzymatically active luciferase after cell-to-cell fusion occurs. We modified this method for use in the HEK293T cell line. FE-H and CA-H cells were parallelly seeded in a 6-well plate (6 × 10^5^ cells/well). After 24 h, cells were cotransfected with 2.4 μg of plasmids expressing either Syncytin-1 or BaEV envelope together with 0.1 μg of pAlpha-SF-mCherry-wPRE to estimate the number of transfected cells. 24 h after transfection, both transfected (FE-H and CA-H) and FE-L and CA-L cells were detached with 1× nonenzymatic cell dissociation solution (Sigma). Corresponding HiBiT and LgBiT cell lines (FE-H+FE-L or CA-H+CA-L) were mixed in ratios 4 × 10^5^:2 × 10^5^ cells, respectively, and transferred to a 6-well plate. After 24 h of incubation, cells were detached with trypsin-ethylenediaminetetraacetic acid (EDTA) solution, resuspended in the fresh culture medium, transferred into 2 mL microtubes, and centrifuged at 300×*g* for 5 min at 4 °C. Cell pellets were resuspended in 350 μL of OptiMEM medium and then transferred per 100 μL in technical triplicates to a whole-white 96-well plate (Nunc) containing 25 µL of Nano-Glo Live Cell Reagent (23.75 µL of LCS Dilution Buffer and 1.25 µL of Live Cell Substrate; Promega) per well, supplemented with 1× DrkBiT peptide (Promega) decreasing the luminescence background in the supernatant. The relative luminescence was measured in a GloMax Explorer luminometer (Promega) after 10 min incubation of the plate at 23 °C and subsequent shaking at 300 RPM for 10 s.

### Heterotrimer Evaluation.

FE-L cells were seeded on 12-well plates, 2 × 10^5^ cells per well. The next day, the cells were transfected with mixtures of the plasmids. On the 1st day posttransfection, the cells were detached with trypsin-EDTA solution, and 1/50 or 1/10 of the suspension was seeded on 96-well plates for infection or expression measurements, respectively. On the 2nd day posttransfection, after the cells attached to the well surface, the cells were infected with Syncytin-1 or BaEV pseudotypes diluted in NP55 to MOI 3, or mock infected with fresh NP55. On the 1st day after infection, the media was exchanged for DMEM55. On the 3rd day after infection, the cells were detached with trypsin-EDTA solution, fixed with paraformaldehyde (1% final concentration), transferred to 96-well U-bottom plates, and analyzed by flow cytometry. The gating strategy is shown in *SI Appendix*, Fig. S11*D*. The expected infection rates shown in Fig. 4*C* were further adjusted to the fraction of transfected cells.

The transfection efficiency was measured by flow cytometry at the same time as the infection was performed. The gating scheme is shown in *SI Appendix*, Fig. S10*A*.

For detection of protein–protein interactions, the FE cells were seeded in duplicates to a whole-white 96-well plate (Nunc) in the concentration of 4 × 10^4^ cells/well and cultivated for 24 h. Cells were transiently cotransfected with homologous or heterologous combinations of human and Chinese hamster ASCT1 or ASCT2 fused either with LgBiT or SmBiT (1:1 ratio for LgBiT:SmBiT). Cotransfections with human EAAT1-LgBiT or -SmBiT were used as a negative control. After 24 h of cultivation, the medium was exchanged for OptiMEM, and 25 µL of Nano-Glo Live Cell Reagent (23.75 µL of LCS Dilution Buffer and 1.25 µL of Live Cell Substrate; Promega) was added per each well. The relative luminescence was measured in a GloMax Explorer luminometer (Promega) after 10 min incubation of the plate at 23 °C and subsequent shaking at 300 RPM for 10 s.

### Statistical Analysis.

For statistical analysis of the intergroup specificity, GraphPad Prism software (version 10.2.3) was employed. Where possible, parametric one-way or two-way ANOVA followed by Dunnett’s or Tukey’s corrections for multiple comparisons was calculated. The data from flow cytometry analysis with non-normal characteristics were evaluated by nonparametric two-tailed Mann–Whitney tests with Holm–Šídák correction for multiple comparisons. In Fig. 3 *E* and *F*, areas under curves for each replicate were calculated and evaluated by the Kruskal–Wallis nonparametric test with Dunn’s correction for multiple comparisons. Calculated P-values were depicted as follows: *** < 0.001, ** < 0.01, * < 0.05, and ns > 0.05.

## Supplementary Material

Appendix 01 (PDF)

## Data Availability

All study data are included in the article and/or *SI Appendix*.
