# Supplementary material for: Receptor usage of Syncytin-1: ASCT2, but not ASCT1, is a functional receptor and effector of cell fusion in the human placenta
Source: Proc Natl Acad Sci U S A. 2024 Oct 21;121(44):e2407519121. doi: 10.1073/pnas.2407519121 (PMC11536146; doi:10.1073/pnas.2407519121)
Supplement: Supplementary file 1 — Appendix 01 (PDF) [file pnas.2407519121.sapp.pdf]

PNAS

Supporting Information for

Receptor usage of Syncytin-1: ASCT2, but not ASCT1, is a functional receptor and effector of cell fusion in the human placenta

Kryštof Štafl, Martin Trávníček, Anna Janovská, Dana Kučerová, Ľubomíra Pecnová, Zhiqi Yang, Vladimír Stepanec, Lukáš Jech, Madhuri S. Salker, Jiří Hejnar, Kateřina Trejbalová

Jiří Hejnar
Email: jiri.hejnar@img.cas.cz

Kateřina Trejbalová
Email: katerina.trejbalova@img.cas.cz

This PDF file includes:

Extended materials and methods
Figures S1 to S13
Table S1
SI References

Extended materials and methods

Generation of ASCT1 and ASCT2 double knockout clones

The guide RNAs (gRNA) for generation of double knockout clones were designed using Crispor (1) and expressed from pX458 vectors, together with Cas9 and EGFP (FPbase ID: R9NL8). The editing of HEK293T cells was performed subsequently. Endogenous *ASCT1* was targeted with gRNA GGCUACCUUGACAGCGCUC. After transfection, singlet cells, expressing Cas9 and EGFP, were separately sorted into 96-well plates, expanded, and screened for the genotype by PCR reaction and Sanger sequencing. Two clones with frameshift mutations, 3F9 and 3C12, were chosen for the second transfection with CRISPR/Cas9 targeting *ASCT2* with gRNA GCGCCUUGGUCCUCGAUGG. The single cell sorting and genotyping were repeated and clones 3F9-2E12 (FE) and 3C12-2A9 (CA) with frameshift mutations were selected.

Mass spectrometry analysis of amino acid uptake

Prior the measurement, the media samples were thawed, shaken and then centrifuged for 20 min, at 15,000 × *g* and 5 °C. 50 µL of supernatant were removed and transferred directly into a 384-well plate (PP Microplate, Echo-MS Qualified, Labcyte). For appropriate well plate sample handling in case of AEMS measurement, well plates were centrifuged at 3500 RPM for 5 min (Multifuge X3R, Thermo Scientific) and then shaken for 2 min at 3500 RPM (Titramax 100, Heidolph Instruments).

MS analysis was performed on AEMS (Echo-MS, TripleQ 6500+, Sciex), where ADE (acoustic droplet ejection) is connected to MS device and the sample is transferred using acoustic ejection from well plate through OPI (open port sampling probe) to the ESI source. Echo-MS is controlled by SciexOS software (3.0) and operated in multiple reaction monitoring (MRM) scan type mode.

MRM transitions for labeled standards (L-Glutamine-2,3,3,4,4-d5, L-Serine-2,3,3-d3, L-Arginine--2,3,3,4,4,5,5-d7; CDN Isotopes) were tuned using guided optimization. For each compound, the transition with the highest intensity and no interference with DMEM/Nutrient Mixture F-12 media was selected (Table S1). For contactless sampling, a total volume of 7.5 nL was ejected in 2.5 nL increments directly into mobile phase (20% MeOH + 0.1% formic acid) at a 400 µL/min flow rate. The ESI source of the triple quadrupole MS instrument was operated in a positive ionization mode with ion source gas 1 set to 90 psi, ion source gas 2 set to 45 psi and curtain gas to 20 psi with the other following MS parameters: ion source temperature 350 °C; spray voltage 5000 V; CAD (collisionally activated dissociation) gas 9 psi.

Concentration of the immunoadhesin

The immunoadhesin was concentrated using an Amicon Ultra centrifugal device with an Ultracel 30000 Dalton molecular weight cutoff filter (Merck, product number UFC9030), following the manufacturer's instructions. 57 mL of the collected supernatant containing the secreted immunoadhesin were loaded onto the device in three portions and centrifuged at 3850 × *g* for a total duration of 50 minutes at 4 °C. The concentration yielded 2.85 mL of immunoadhesin, resulting in a volume reduction of 20-fold.

Proteomic mass spectrometry analysis of precipitated proteins

About 100 million of CA-L/hsASCT1 and HEK-L cells were washed twice with 10 mL of PBS and scraped off in 1× lysis buffer (1% Brij 97, 150 mM NaCl, 20 mM Tris pH 8, 5 mM iodacetamid). Cells were rotated for 1 hour at 4 °C to lyse and centrifuged for 10 minutes, 5000 × *g*, 4 °C. The immunoadhesin was bound to the protein G-agarose (PierceTM Protein G Agarose, ThermoFisher Scientific, catalog number 20398) and incubated with cell lysates overnight at 4 °C. Protein G-agarose beads with bound proteins were centrifuged (5000 × *g*, 2 min) and washed two times with the cold lysis buffer and three times with the cold PBS.

For sample preparation, on-bead digestion protocol was used. Briefly, washed beads were resuspended in sodium deoxycholate (SDC, final concentration 1% [w/v] in 100 mM TEAB, triethylammonium bicarbonate), reduced with 5 mM TCEP [tris(2-carboxyethyl)phosphine], alkylated with 20 mM chloroacetamide and digested overnight with trypsin. SDC was removed by extraction with ethylacetate saturated with water (2). Samples were desalted on Empore C18

columns, dried in a speedvac, and dissolved in 0.1% TFA + 2% acetonitrile. Desalted peptide digests were separated on a 50 cm C18 column using 60 min elution gradient and analyzed in a DDA mode on an Orbitrap Exploris 480 (Thermo Fisher Scientific) mass spectrometer equipped with the FAIMS unit operated at -40 and -60 V CVs.

The resulting raw files were converted to mzXML files each containing separate compensation voltage (CV, -40, -60 V) scans using FAIMS MzXML Generator (release 1.1.8003, https://github.com/PNNL-Comp-Mass-Spec/FAIMS-MzXML-Generator). MzXML files were analyzed in MaxQuant (v. 2.4.7.0) with label-free quantification (LFQ) algorithm MaxLFQ and match between runs feature activated. FDR was set as 0.01 at all levels. UniProt human UP000005640_9606.fasta release 2023_01 was used. Carbamidomethyl cysteine was selected as a fixed modification, variable modifications were Oxidation (M) and Acetyl (Protein N-term). Downstream processing of the proteinGroups.txt file was performed in Perseus (v2.0.11).

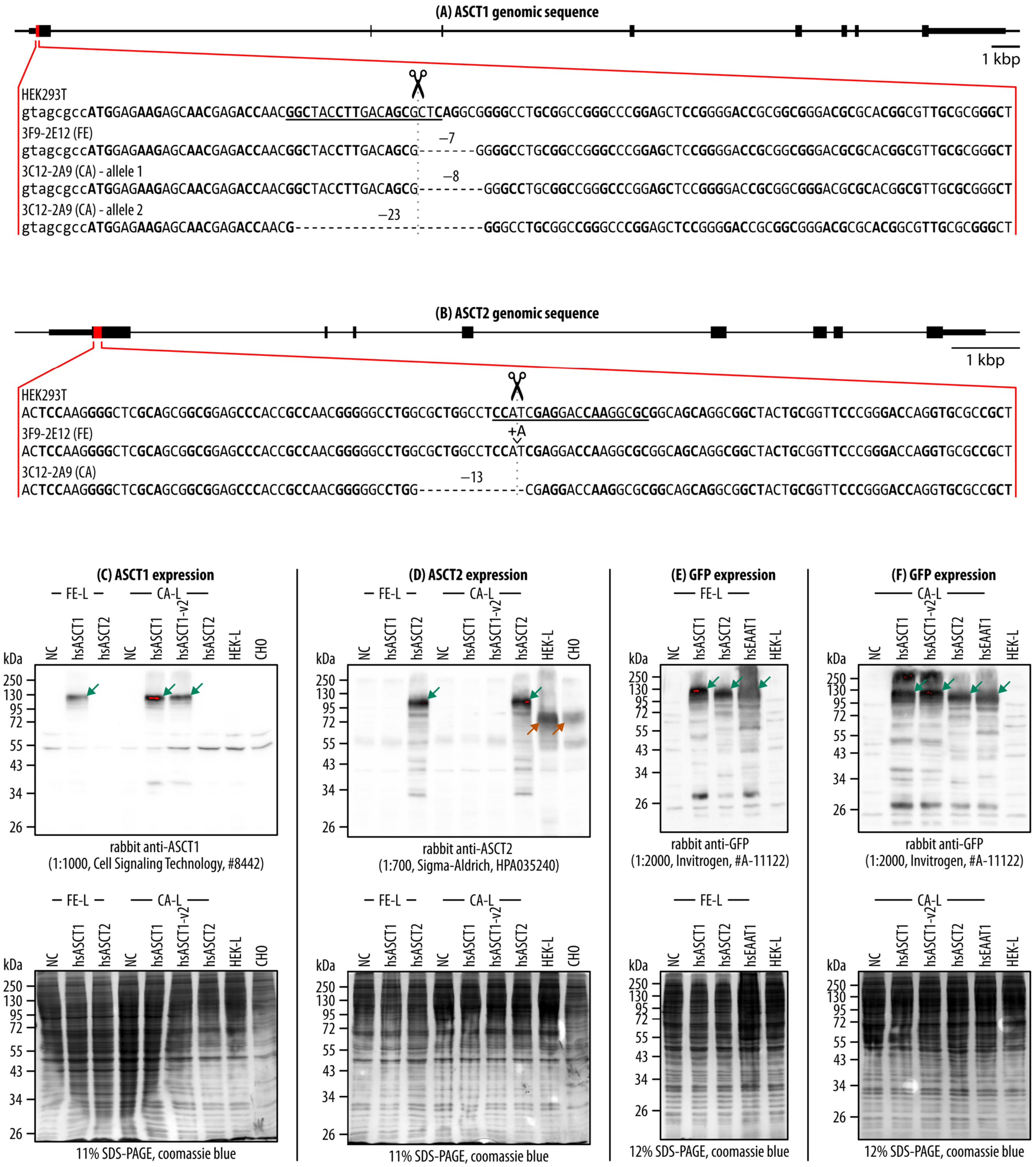

(A) ASCT1 genomic sequence
1 kbp
HEK293T
gtagcgccATGGAGAAGAGCAACGAGACCAACGGCTACCTTGACAGCGCTCAGGCGGGGCCTGCGGCCGGGCCCGGAGCTCCGGGGACCGCGGCGGGACGCGCACGGCGTTGCGCGGGCT
3F9-2E12 (FE)
−7
gtagcgccATGGAGAAGAGCAACGAGACCAACGGCTACCTTGACAGCG-------GGGGCCTGCGGCCGGGCCCGGAGCTCCGGGGACCGCGGCGGGACGCGCACGGCGTTGCGCGGGCT
3C12-2A9 (CA) - allele 1
−8
gtagcgccATGGAGAAGAGCAACGAGACCAACGGCTACCTTGACAGCG--------GGGCCTGCGGCCGGGCCCGGAGCTCCGGGGACCGCGGCGGGACGCGCACGGCGTTGCGCGGGCT
3C12-2A9 (CA) - allele 2
−23
gtagcgccATGGAGAAGAGCAACGAGACCAACG-----------------------GGGCCTGCGGCCGGGCCCGGAGCTCCGGGGACCGCGGCGGGACGCGCACGGCGTTGCGCGGGCT
(B) ASCT2 genomic sequence
1 kbp
HEK293T
ACTCCAAGGGGCTCGCAGCGGCGGAGCCCACCGCCAACGGGGGCCTGGCGCTGGCCTCCATCGAGGACCAAGGCGCGGCAGCAGGCGGCTACTGCGGTTCCCGGGACCAGGTGCGCCGCT
3F9-2E12 (FE)
+A
ACTCCAAGGGGCTCGCAGCGGCGGAGCCCACCGCCAACGGGGGCCTGGCGCTGGCCTCCATCGAGGACCAAGGCGCGGCAGCAGGCGGCTACTGCGGTTCCCGGGACCAGGTGCGCCGCT
3C12-2A9 (CA)
−13
ACTCCAAGGGGCTCGCAGCGGCGGAGCCCACCGCCAACGGGGGCCTGG-------------CGAGGACCAAGGCGCGGCAGCAGGCGGCTACTGCGGTTCCCGGGACCAGGTGCGCCGCT
(C) ASCT1 expression
FE-L
CA-L
NC
hsASCT1
hsASCT2
NC
hsASCT1
hsASCT1-v2
hsASCT2
HEK-L
CHO
kDa
250
130
95
72
55
43
34
26
rabbit anti-ASCT1
(1:1000, Cell Signaling Technology, #8442)
11% SDS-PAGE, coomassie blue
(D) ASCT2 expression
FE-L
CA-L
NC
hsASCT1
hsASCT2
NC
hsASCT1
hsASCT1-v2
hsASCT2
HEK-L
CHO
kDa
250
130
95
72
55
43
34
26
rabbit anti-ASCT2
(1:700, Sigma-Aldrich, HPA035240)
11% SDS-PAGE, coomassie blue
(E) GFP expression
FE-L
NC
hsASCT1
hsASCT2
hsEAAT1
HEK-L
kDa
250
130
95
72
55
43
34
26
rabbit anti-GFP
(1:2000, Invitrogen, #A-11122)
12% SDS-PAGE, coomassie blue
(F) GFP expression
CA-L
NC
hsASCT1
hsASCT1-v2
hsASCT2
hsEAAT1
HEK-L
kDa
250
130
95
72
55
43
34
26
rabbit anti-GFP
(1:2000, Invitrogen, #A-11122)
12% SDS-PAGE, coomassie blue

Fig. S1. CRISPR/Cas9 editing of *ASCT1* and *ASCT2* and ectopic expression of the transporters. (A-B) Schematic depiction of human *ASCT1* and *ASCT2* genomic organization, boxes correspond to exons, coding sequence is depicted in bold. The targeted sequence is underlined and the scissors with dotted line point to the Cas9 cleavage sites. Genomic sequences of HEK293T and selected FE and CA clones were determined by Sanger sequencing. The size of frameshift deletion is shown as number; clone FE acquired homozygous frameshift in *ASCT1* and a homozygous single nucleotide insertion into *ASCT2*. CA clone was heterozygous for *ASCT1* with both alleles depicted but was homozygous for *ASCT2* frameshift. Coding sequences are shown in upper-case; the codons are distinguished by bold font. **(C-F)** Western blot analysis of protein expression of HEK-L, CHO, and double knockout clones with or without (NC) ectopic expression of human transporters. Brown arrows point to wild-type transporters (anti-ASCT1, anti-ASCT2 antibodies, expected migration rate around 80 kDa), green arrows to transporters fused with GFP (anti-ASCT1, anti-ASCT2, anti-GFP antibodies, expected migration rate around 110 kDa). HEK-L cells do not express endogenous ASCT1. As a loading control, the gels were stained with Coomassie blue after the separated proteins were transferred to membrane.

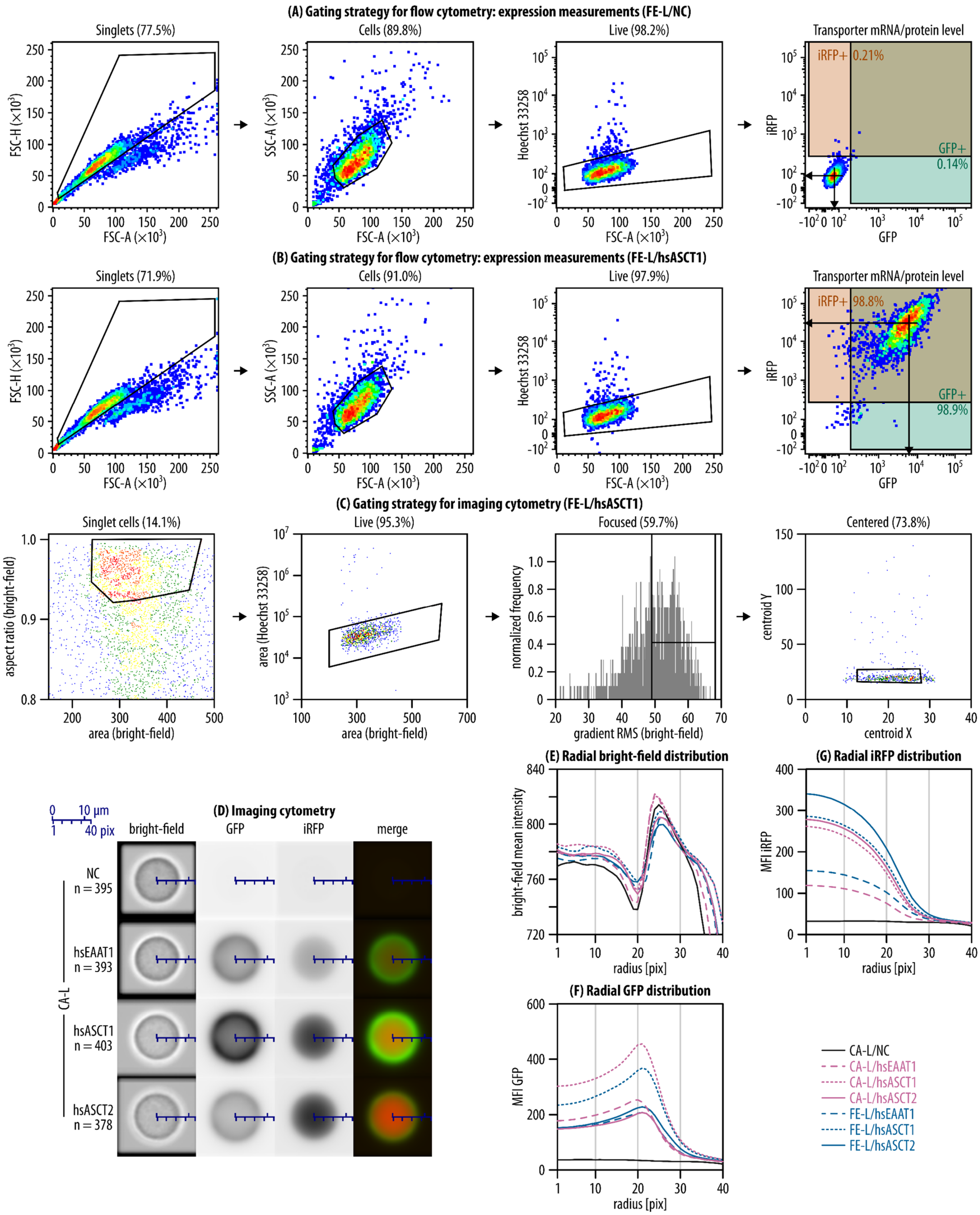

(A) Gating strategy for flow cytometry: expression measurements (FE-L/NC)
Singlets (77.5%)
Cells (89.8%)
Live (98.2%)
Transporter mRNA/protein level
iRFP+ 0.21%
GFP+ 0.14%
(B) Gating strategy for flow cytometry: expression measurements (FE-L/hsASCT1)
Singlets (71.9%)
Cells (91.0%)
Live (97.9%)
Transporter mRNA/protein level
iRFP+ 98.8%
GFP+ 98.9%
(C) Gating strategy for imaging cytometry (FE-L/hsASCT1)
Singlet cells (14.1%)
Live (95.3%)
Focused (59.7%)
Centered (73.8%)
(D) Imaging cytometry
bright-field
GFP
iRFP
merge
NC n = 395
hsEAAT1 n = 393
hsASCT1 n = 403
hsASCT2 n = 378
CA-L
(E) Radial bright-field distribution
(F) Radial GFP distribution
(G) Radial iRFP distribution
CA-L/NC
CA-L/hsEAAT1
CA-L/hsASCT1
CA-L/hsASCT2
FE-L/hsEAAT1
FE-L/hsASCT1
FE-L/hsASCT2

Fig. S2. Gating schemes for flow and imaging cytometry of live cells. (A-B) Representative flow cytometry gating strategy for Fig. 1B-E. 4×10^4 of the cells were seeded on 96-well plates. On the next day, the cells were washed by PBS, detached with trypsin-EDTA solution and transferred to 96-well plate with U-shaped bottom. The cellular suspension was pelleted by 3-minutes centrifugation with 450 × *g*. The pellets were resuspended in 50 µl of PBS with Hoechst 33258 (1 µg per mL) and characterized by FACSymphony (BD) flow cytometer equipped with high throughput sampler. Live cells were defined as follows: i. singlet events according to their ratio of area to height in forward scatter (FSC-A, FSC-H); ii. cells according to their area in forward scatter (FSC-A) and side scatter (SSC-A); iii. live cells unstained by Hoechst 33258 (excitation 405 nm, emission 450/50 nm). The resulting populations were further analyzed for fractions of iRFP (ex. 637 nm, em. 730/45 nm) and GFP (ex. 488 nm, em. 530/30 nm) expressing cells (overlapping rectangle gates) and mean fluorescent intensity (arrows in right dot plots). The analyses were performed in FlowJo software (v10.10) and the frequency of parent (%) is shown. **(C)** Representative imaging cytometry gating strategy for Fig. 1F. Three million of the cells were seeded on P100-dishes. On the next day, the cells were washed by PBS and detached with trypsin-EDTA solution. The cellular suspension was pelleted by 8-minutes centrifugation with 200 × *g*. The pellet (approx. 20 µl) was resuspended in 100 µl of PBS with Hoechst 33258 (10 µg per mL). The sample was analyzed with Amnis ImageStreamX Mk II (Cytek) imaging flow cytometer in 60× magnification, resulting in pixelsize of 0.3×0.3 µm. The analyzed cells were defined as follows: i. singlet cells according to the aspect ratio and area of mask in the bright-field channel; ii. live cells unstained by Hoechst 33258 (ex. 405 nm, em. 457/45 nm); iii. focused cells according to microcontrast in the bright-field channel; iv. centered cells by their bright-field mask coordinates. The analysis was performed in IDEAS software (6.2) and the frequency of parent (%) is shown. **(D)** Localization of ectopically expressed transporters throughout the CA-L cells by imaging cytometry. Raw images from (C) were exported and analyzed with Fiji/ImageJ. First, the images were aligned to the canvas center by adjusting their bright-field mask centroid position. Second, the pixel intensities in channels of bright-field, GFP (ex. 488 nm, em. 528/65 nm), and iRFP (ex. 642 nm, em. 702/85 nm) of all images of the same cell type were averaged. The minimum and maximum pixel values of images were determined; all images were linearly adjusted according to that and converted to 8-bit pictures. The averaged images for individual transporters are shown in bright-field, GFP, and iRFP channels, and as composites of the GFP (green) and iRFP channels (red). The blue scale bars show radius in pixels and µm; n represents the number of averaged cells. **(E-G)** The summarized radial signal distribution of the average images from Fig. S2D was calculated with the Radial Profile Extended plugin (http://questpharma.u-strasbg.fr/html/radial-profile-ext.html) and plotted. The contrast transition in bright-field **(E)** points to the cellular edge. GFP **(F)** and iRFP **(G)** channels demonstrate cellular localization of transduced proteins.

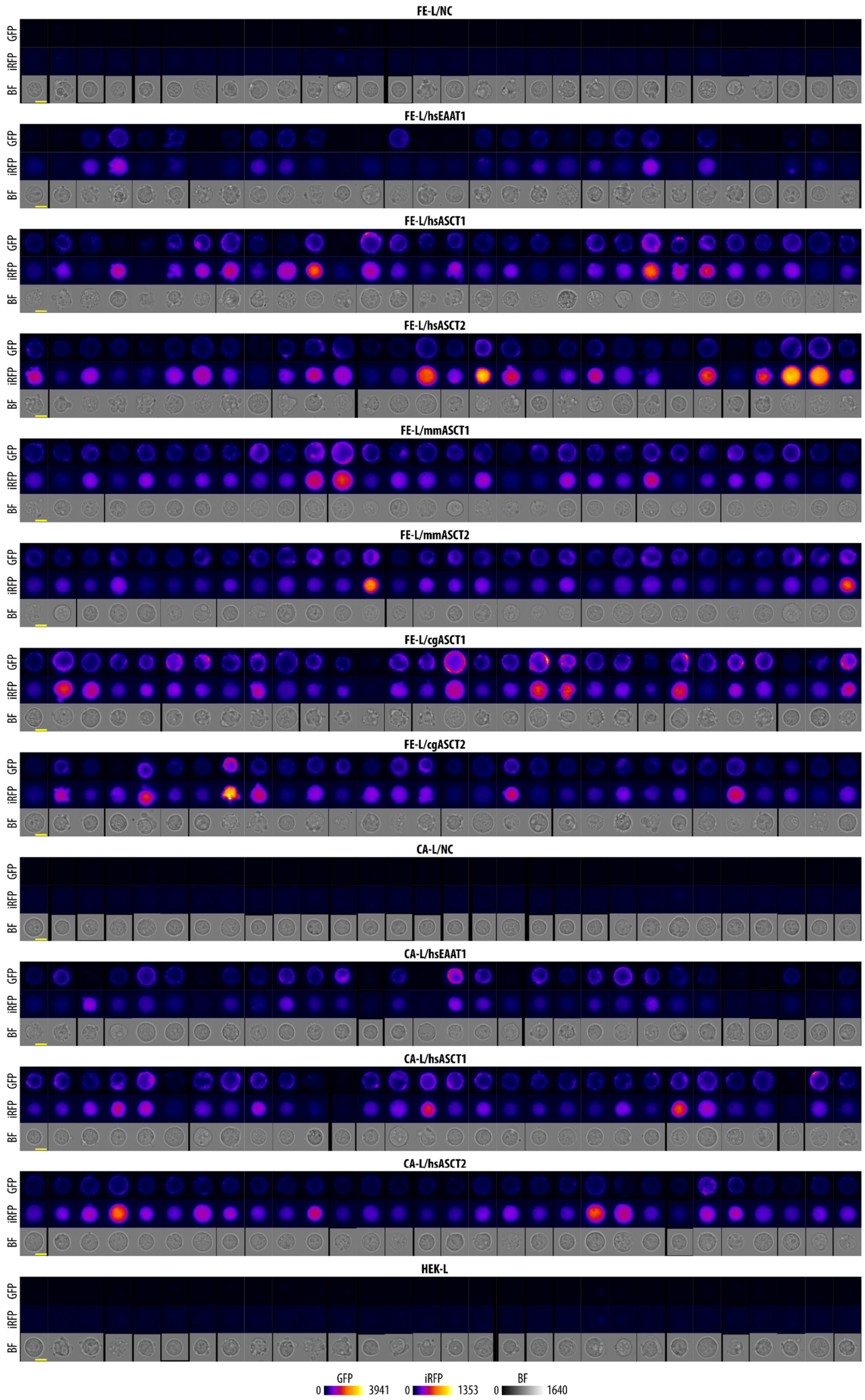
FE-L/NC
FE-L/hsEAAT1
FE-L/hsASCT1
FE-L/hsASCT2
FE-L/mmASCT1
FE-L/mmASCT2
FE-L/cgASCT1
FE-L/cgASCT2
CA-L/NC
CA-L/hsEAAT1
CA-L/hsASCT1
CA-L/hsASCT2
HEK-L
GFP
iRFP
BF
0
3941
1353
1640

Fig. S3. Representative images of cells acquired by imaging cytometry. Images of focused cells were aligned and pseudo-colorized in Fiji/ImageJ software. Signal localizations of GFP, iRFP and bright-field (BF) are shown. These images are a representative part showing 30 cells out of source data used for analysis in Fig. 1F and Fig. S2D. Gating strategy is shown in Fig. S2C. NC, negative control, FE-L or CA-L cells with no ectopic expression. Yellow scale bars represent 10 μm.

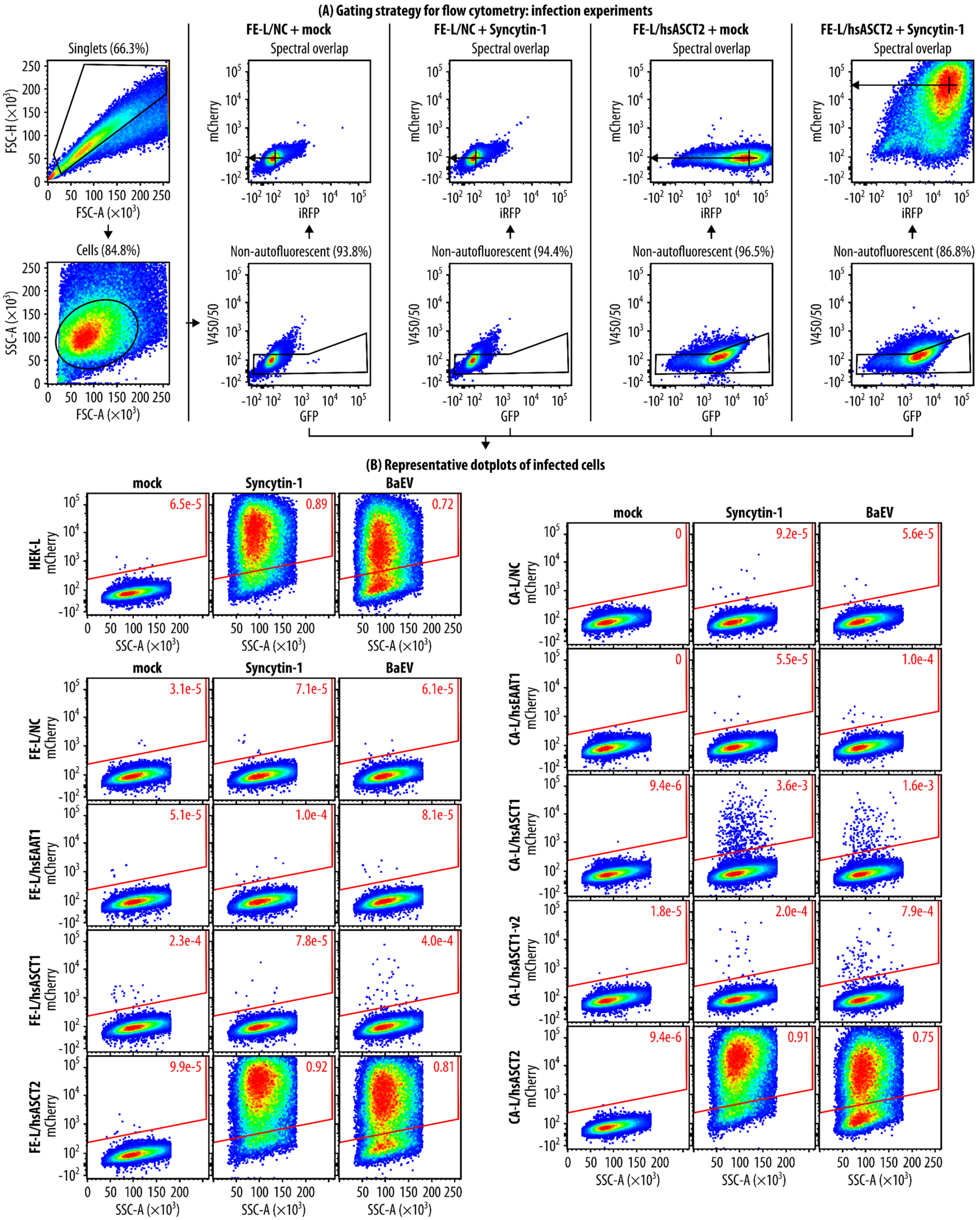

(A) Gating strategy for flow cytometry: infection experiments
Singlets (66.3%)
FSC-H (×10³)
FSC-A (×10³)
Cells (84.8%)
SSC-A (×10³)
FE-L/NC + mock
FE-L/NC + Syncytin-1
FE-L/hsASCT2 + mock
FE-L/hsASCT2 + Syncytin-1
Spectral overlap
mCherry
iRFP
Non-autofluorescent (93.8%)
Non-autofluorescent (94.4%)
Non-autofluorescent (96.5%)
Non-autofluorescent (86.8%)
V450/50
GFP
(B) Representative dotplots of infected cells
mock
Syncytin-1
BaEV
HEK-L
6.5e-5
0.89
0.72
FE-L/NC
3.1e-5
7.1e-5
6.1e-5
FE-L/hsEAAT1
5.1e-5
1.0e-4
8.1e-5
FE-L/hsASCT1
2.3e-4
7.8e-5
4.0e-4
FE-L/hsASCT2
9.9e-5
0.92
0.81
CA-L/NC
0
9.2e-5
5.6e-5
CA-L/hsEAAT1
0
5.5e-5
1.0e-4
CA-L/hsASCT1
9.4e-6
3.6e-3
1.6e-3
CA-L/hsASCT1-v2
1.8e-5
2.0e-4
7.9e-4
CA-L/hsASCT2
9.4e-6
0.91
0.75

Fig. S4. Flow cytometry of infected cells. (A) Gating scheme for infection experiments. The cells were detached with trypsin, fixed in paraformaldehyde (1% final concentration), transferred to 96-well plate with U-shaped bottom and characterized by FACSymphony (BD) flow cytometer equipped with high throughput sampler. Cells were defined as follows: i. singlet events according to their ratio of area to height in forward scatter (FSC-A, FSC-H); ii. cells according to their area in forward scatter (FSC-A) and side scatter (SSC-A); iii. non-autofluorescent cells in V450/50 channel (excitation 405 nm, emission 450/50 nm). The analyses were performed in FlowJo software (v10.10) and the frequency of parent (%) is shown. No spillover of iRFP fluorescence (ex. 637 nm, em. 730/45 nm) to mCherry channel (ex. 561 nm, em. 610/20 nm) was detected. **(B)** The infected cells from (A) were defined by their signal in SSC-A and mCherry channels. The red numbers correspond to fractions of cells in the red quadrangles. Representative dotplots are shown. NC, negative control, FE-L or CA-L cells with no ectopic expression.

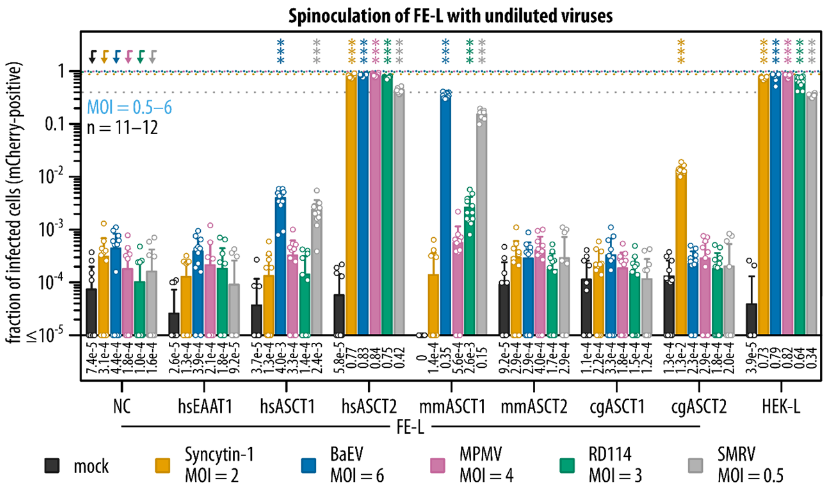

Fig. S5. Evaluation of ASCT1 and ASCT2 receptor function for viruses of the RDR interference group. Double knockout clones FE-L expressing different transporters were spinoculated with undiluted pseudotypes of Syncytin-1 (MOI 2), BaEV (MOI 6), MPMV (MOI 4), RD114 (MOI 3) or SMRV (MOI 0.5) or mock spinoculated. The pseudotypes transduced mCherry fluorescent marker, the fractions of mCherry-positive cells represent fractions of infected cells. Data are shown as means with standard deviation; the means are also reported as numerical values below the bars. Dotted lines indicate expected infection rates. Mann-Whitney non-parametric statistical tests with Holm-Šídák multiple comparison correction were performed, *P* values related to arrow-pointed controls are following: <0.001: ***. The total number of biological replicates is shown as n. The gating scheme is shown in Fig. S4A. NC, negative control, FE-L cells with no ectopic expression.

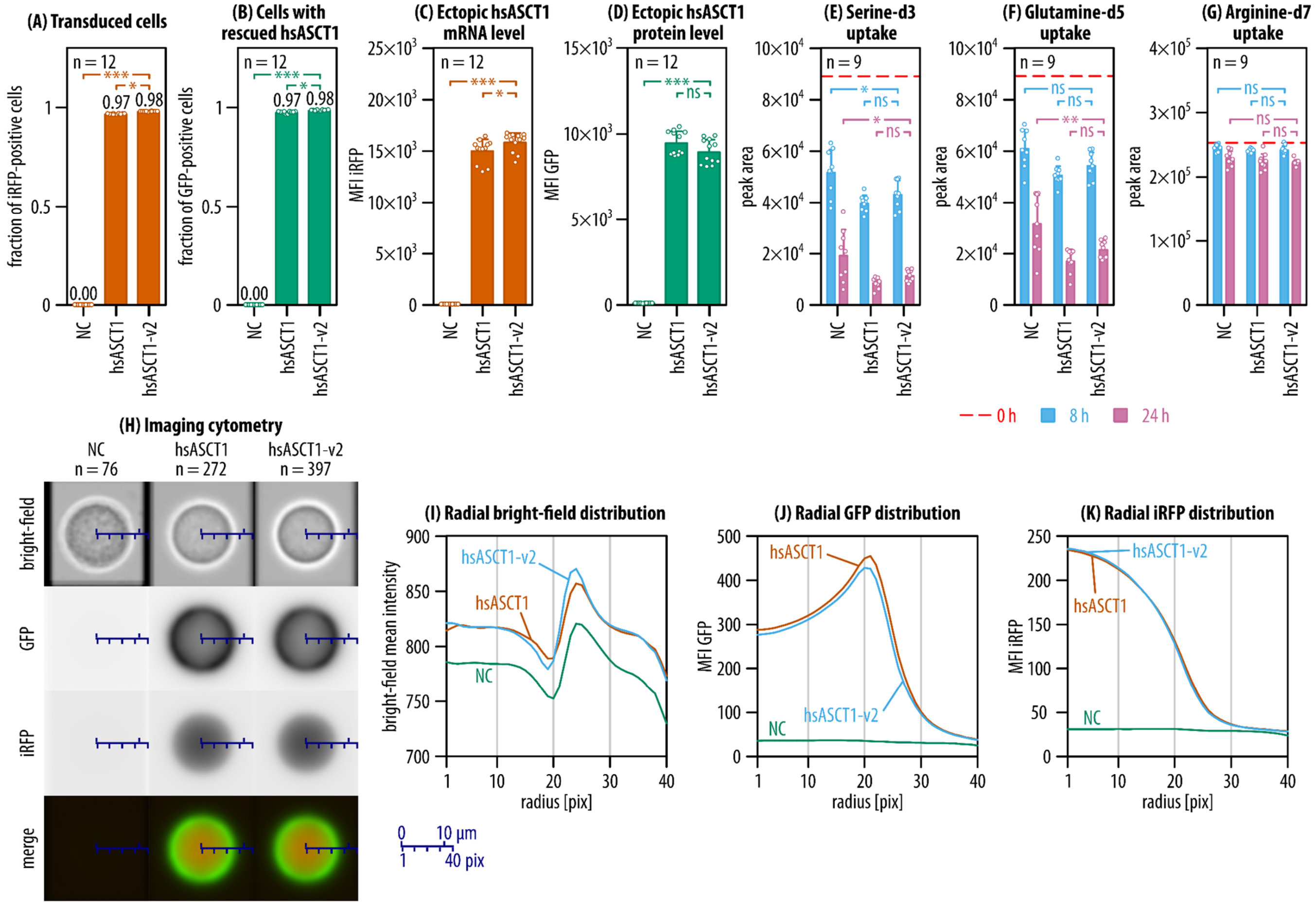

Fig. S6. Comparison of CA-L/hsASCT1-v2 with the CA-L/hsASCT1 and negative control. (A-D) Flow cytometry analysis of the purity after sorting and expression levels of hsASCT1. iRFP corresponds to levels of hsASCT1 mRNA, GFP corresponds to levels of hsASCT1 protein. **(E-G)** Uptake of deuterated serine, glutamine or arginine was measured after 8 or 24 hours. The red dashed lines indicate the initial amounts of the labeled amino acids in the medium, and the blue and pink bars show their decrease after 8 or 24 hours of cultivation, respectively. The measurement of CA-L/hsASCT1-v2 was performed together with the samples shown in Figure 1J-L. **(H)** Imaging cytometry analysis of hsASCT1 localization in cells. Images of focused cells were aligned, and average intensities in bright-field, GFP, and iRFP channels are shown, together with composites of the GFP (green) and iRFP channels (red). The scale bars show radius in pixels and µm. **(I-K)** The radial signal distribution of each channel was further analyzed and plotted. The contrast transition in bright-field points to the cellular edge. All bars are shown as means with standard deviation. Statistical tests: Kruskal-Wallis with Dunn's multiple comparison correction (A-B), one-way ANOVA with Dunnett's correction (C-D), two-way ANOVA with Tukey's correction (E-G). *P* values as follows: >0.05: ns, <0.05: *, <0.01: **, <0.001: ***. The experiments in A-D were repeated four times, experiments in E-G three times. The numbers of total biological replicates in A-G are shown as n. In H, n corresponds to the number of analyzed and averaged cells. NC, negative control, CA-L cells with no ectopic expression. MFI, mean fluorescence intensity.

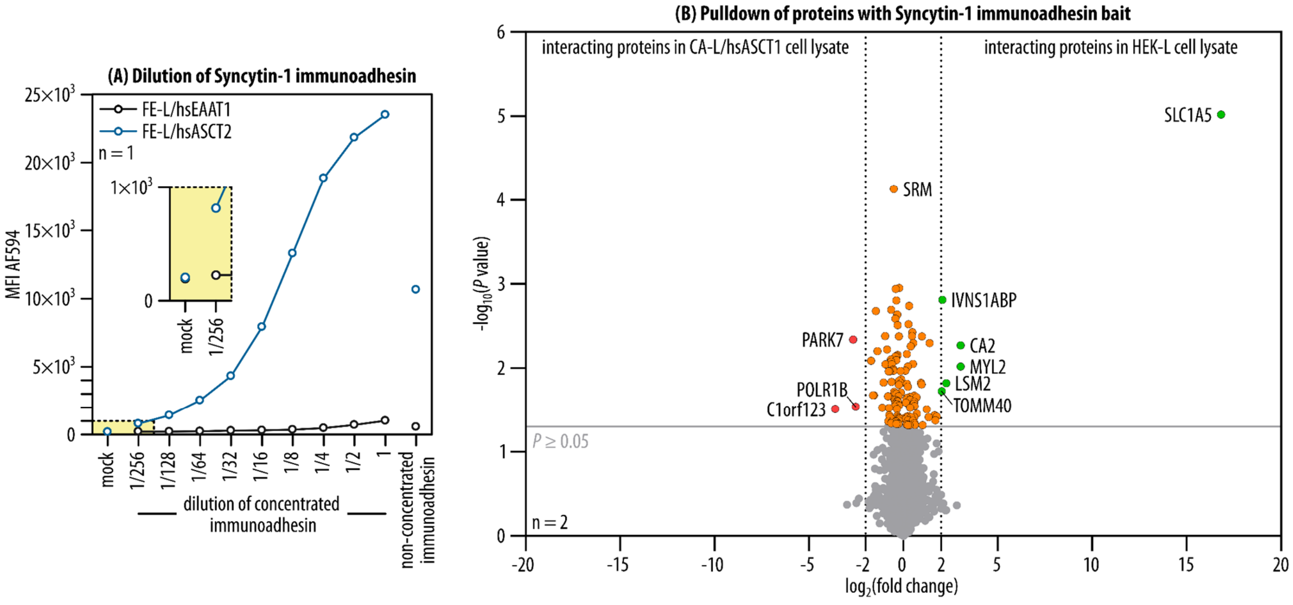

Fig. S7. Syncytin-1 immunoadhesin specificity. (A) The specificity of Syncytin-1 immunoadhesin binding to hsASCT2 characterized by the immunoadhesin dilution. FE-L/hsEAAT1 and FE-L/hsASCT2 were used for the immunoadhesin-binding assay. The concentrated Syncytin-1 immunoadhesin (see Extended materials and methods) was diluted 2-, 4-, 8-, 16-, 32-, 64-, 128-, and 256-times. The non-concentrated immunoadhesin was used as a reference. In mock controls, only the anti-IgG antibody conjugated with Alexa Fluor 594 was used for labeling. The non-specific labeling of the anti-IgG antibody is shown in the enlarged yellow inset. The experiment was performed in technical duplicates, mean of MFI is depicted. **(B)** The specificity of Syncytin-1 immunoadhesin binding to hsASCT2 characterized by proteomic analysis. Syncytin-1 immunoadhesin was used as a bait to immunoprecipitate the whole cell lysates of the HEK-L cells and CA-L/hsASCT1. See Extended methods for a detailed description. HEK-L cells do not express the endogenous ASCT1 protein (Fig. S1C) but do express the ASCT2 protein (Fig. S1D). Precipitated proteins bound by the Syncytin-1 immunoadhesin and identified by proteomic analysis are depicted. Samples were run in duplicates.

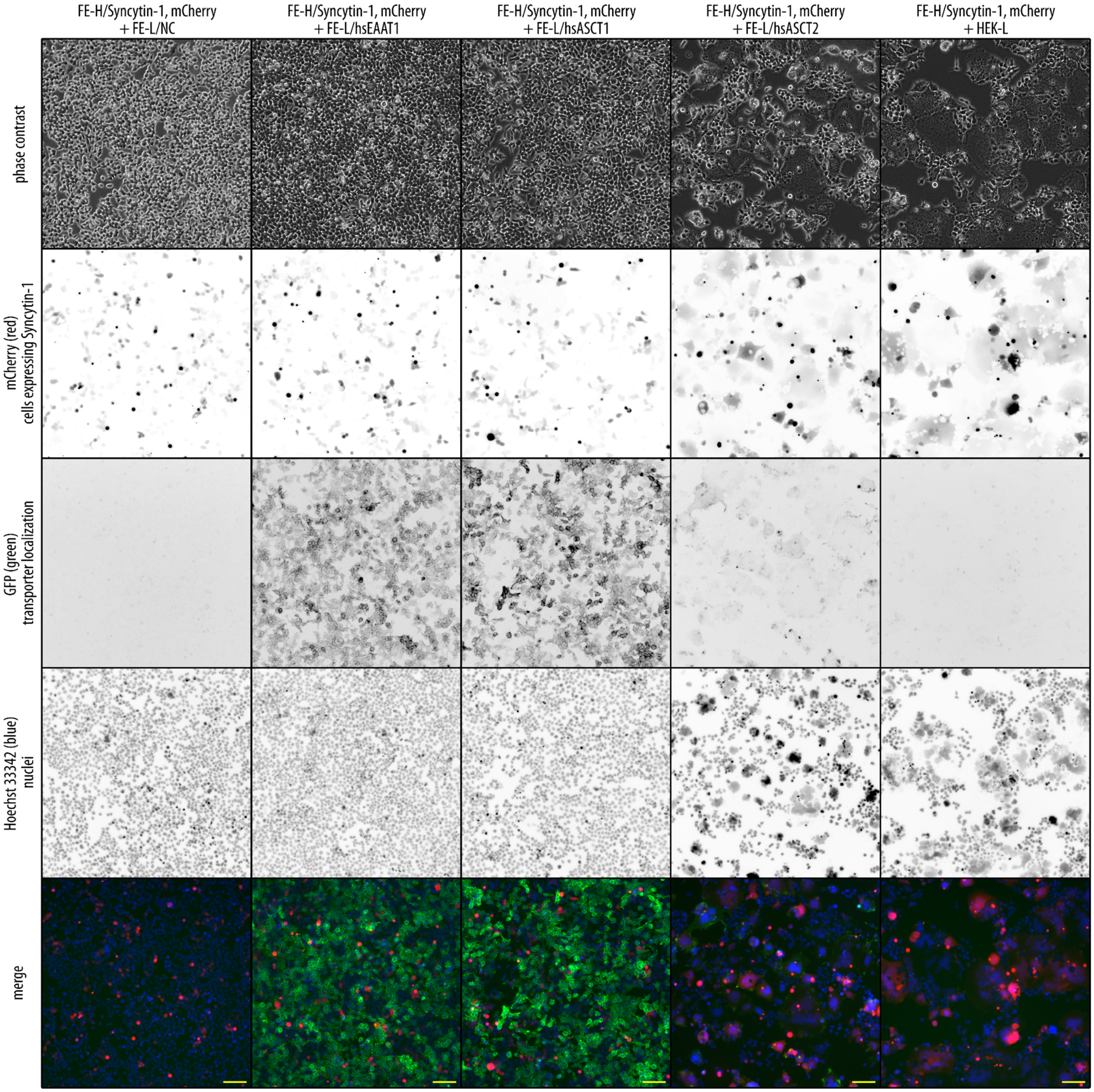

Fig. S8. Syncytium formation. 4×10[5] of FE-H cells, cotransfected with plasmids encoding Syncytin-1 and mCherry, were cocultivated with 4×10[5] FE-L cells ectopically expressing different transporters, or HEK-L cells. Two days post transfection, the cells were stained with Hoechst 33342 and visualized with Nikon Ti2 inverted fluorescent microscope with 10× objective and DS-10 camera. Images are separately shown in bright-field and fluorescent channels and also as fluorescent composites. mCherry channel (red) shows cells expressing Syncytin-1, GFP channel (green) shows the localization of GFP-tagged transporters, Hoechst channel (blue) shows cell nuclei. The scale bars correspond to 100 µm. NC, negative control, FE-L cells with no ectopic expression.

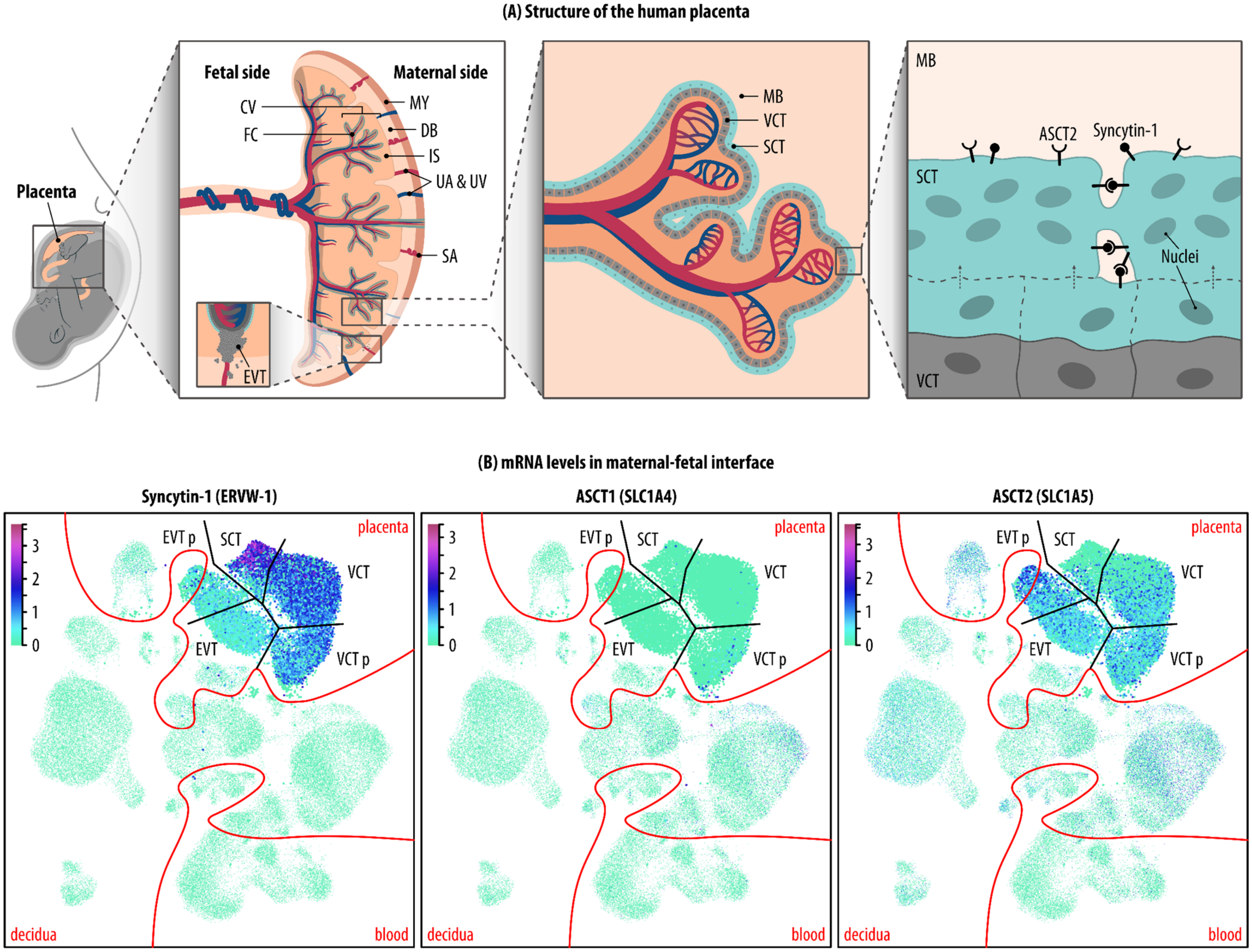

Fig. S9. Maternal-fetal interface. (A) A schematic representation of the human placenta structure. Left: a fully developed human placenta with spiral arteries delivering blood to the intervillous space. Middle: mature chorionic villi with multinucleated syncytiotrophoblast as the outer layer (turquoise). Right: the development of syncytiotrophoblast from cytotrophoblast through Syncytin-1 and ASCT2--mediated fusion. **(B)** Single-cell RNA sequencing data of *Syncytin-1*, *ASCT1* and *ASCT2* expression at maternal-fetal interface. Analysis of publicly available data published by Vento-Tormo *et al*. (3, https://maternal-fetal-interface.cellgeni.sanger.ac.uk/) visualized with CZ CELLxGENE Explorer (4). The scale corresponds to the logarithm of normalized expression. CV – chorionic villus, FC – fetal capillary, MY – myometrium, DB – *decidua basalis*, IS – intervillous space, UA – uterine artery, UV – uterine vein, SA – spiral artery, EVT – extravillous trophoblast, MB – mother's blood, VCT – villous cytotrophoblast, SCT – syncytiotrophoblast, p – proliferative.

(A) Gating strategy: coexpression of tranposrters

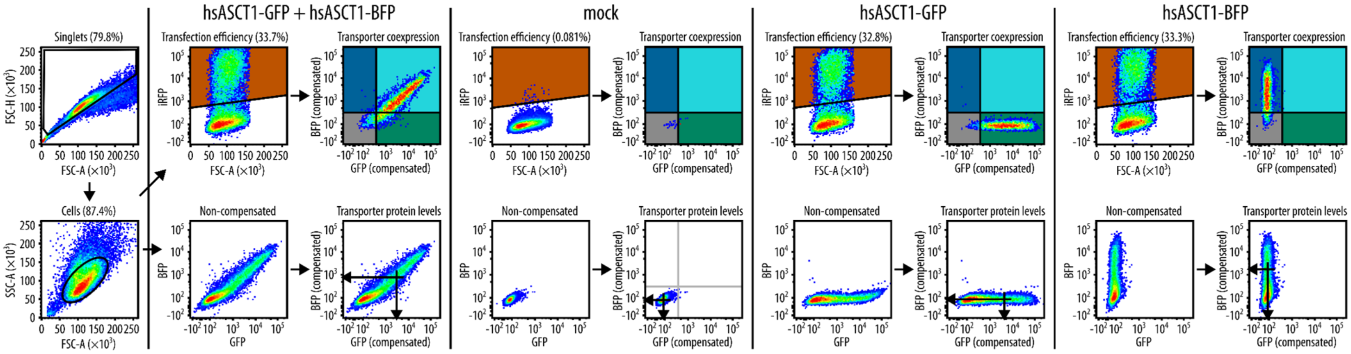

(B) Transfection efficiency (fraction of iRFP-positive ±RSD)

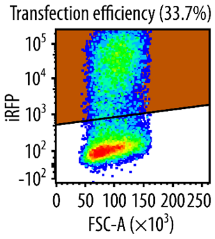

	mock-transfected	GFP: hsEAAT1	GFP: hsASCT1	GFP: hsASCT2	GFP: cgASCT1	GFP: cgASCT2
	7.2e-5 ±14% (n = 3)					
single transfection		0.31 ±15% (n = 3)	0.34 ±11% (n = 3)	0.31 ±14% (n = 3)	0.29 ±16% (n = 3)	0.32 ±11% (n = 3)
BFP: hsEAAT1	0.34 ±12% (n = 3)	0.32 ±8% (n = 3)	0.35 ±6% (n = 3)	0.34 ±12% (n = 3)	0.34 ±8% (n = 3)	0.32 ±11% (n = 3)
BFP: hsASCT1	0.35 ±8% (n = 3)	0.32 ±10% (n = 3)	0.34 ±10% (n = 3)	0.33 ±12% (n = 3)	0.33 ±12% (n = 3)	0.33 ±12% (n = 3)
BFP: hsASCT2	0.38 ±5% (n = 2)	0.32 ±7% (n = 3)	0.37 ±9% (n = 2)	0.32 ±12% (n = 3)	0.33 ±10% (n = 3)	0.31 ±13% (n = 3)
BFP: cgASCT1	0.35 ±10% (n = 3)	0.34 ±4% (n = 3)	0.35 ±11% (n = 3)	0.32 ±17% (n = 3)	0.31 ±13% (n = 3)	0.32 ±2% (n = 3)
BFP: cgASCT2	0.35 ±14% (n = 3)	0.31 ±5% (n = 3)	0.36 ±6% (n = 3)	0.34 ±10% (n = 3)	0.33 ±7% (n = 3)	0.33 ±11% (n = 3)

(C) Transporter protein level (GFP-MFI ±RSD)

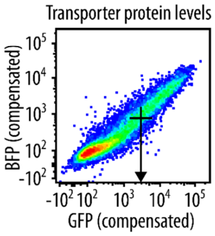

	mock-transfected	GFP: hsEAAT1	GFP: hsASCT1	GFP: hsASCT2	GFP: cgASCT1	GFP: cgASCT2
	86 ±1% (n = 3)					
single transfection		2011 ±49% (n = 3)	3609 ±47% (n = 3)	1338 ±40% (n = 3)	2667 ±44% (n = 3)	1366 ±45% (n = 3)
BFP: hsEAAT1	90 ±1% (n = 3)	1322 ±44% (n = 3)	3069 ±46% (n = 3)	1215 ±38% (n = 3)	2746 ±43% (n = 3)	1187 ±40% (n = 3)
BFP: hsASCT1	91 ±5% (n = 3)	996 ±48% (n = 3)	2106 ±44% (n = 3)	663 ±35% (n = 3)	1695 ±45% (n = 3)	751 ±39% (n = 3)
BFP: hsASCT2	89 ±1% (n = 2)	1052 ±41% (n = 3)	3400 ±8% (n = 2)	883 ±39% (n = 3)	2107 ±40% (n = 3)	907 ±44% (n = 3)
BFP: cgASCT1	89 ±1% (n = 3)	1086 ±42% (n = 3)	2628 ±47% (n = 3)	832 ±40% (n = 3)	1755 ±43% (n = 3)	932 ±39% (n = 3)
BFP: cgASCT2	93 ±4% (n = 3)	1152 ±41% (n = 3)	2823 ±47% (n = 3)	1037 ±38% (n = 3)	2190 ±49% (n = 3)	977 ±44% (n = 3)

(D) Transporter protein level (BFP-MFI ±RSD)

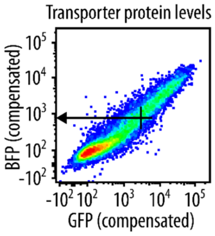

	mock-transfected	GFP: hsEAAT1	GFP: hsASCT1	GFP: hsASCT2	GFP: cgASCT1	GFP: cgASCT2
	89 ±10% (n = 3)					
single transfection		92 ±5% (n = 3)	90 ±10% (n = 3)	89 ±9% (n = 3)	93 ±7% (n = 3)	94 ±5% (n = 3)
BFP: hsEAAT1	1536 ±41% (n = 3)	440 ±23% (n = 3)	327 ±24% (n = 3)	366 ±32% (n = 3)	374 ±16% (n = 3)	355 ±28% (n = 3)
BFP: hsASCT1	1573 ±29% (n = 3)	1009 ±31% (n = 3)	660 ±30% (n = 3)	718 ±34% (n = 3)	803 ±23% (n = 3)	835 ±31% (n = 3)
BFP: hsASCT2	766 ±2% (n = 2)	365 ±16% (n = 3)	253 ±9% (n = 2)	278 ±12% (n = 3)	248 ±19% (n = 3)	276 ±17% (n = 3)
BFP: cgASCT1	1270 ±28% (n = 3)	756 ±23% (n = 3)	473 ±29% (n = 3)	551 ±31% (n = 3)	495 ±15% (n = 3)	615 ±16% (n = 3)
BFP: cgASCT2	938 ±35% (n = 3)	454 ±27% (n = 3)	288 ±21% (n = 3)	320 ±24% (n = 3)	347 ±8% (n = 3)	338 ±20% (n = 3)

(E) Transporter coexpression in iRFP-positive cells (percent of GFP+BFP double positive in transfected cells)

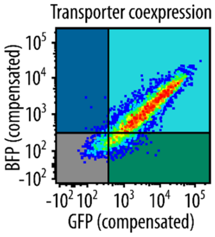

		GFP: hsEAAT1	GFP: hsASCT1	GFP: hsASCT2	GFP: cgASCT1	GFP: cgASCT2
single transfection						
BFP: hsEAAT1		55%	49%	49%	52%	48%
BFP: hsASCT1		66%	67%	62%	72%	60%
BFP: hsASCT2		49%	42%	44%	40%	42%
BFP: cgASCT1		66%	61%	65%	66%	63%
BFP: cgASCT2		52%	42%	45%	48%	46%

Fig. S10. Cotransfection of GFP- and BFP-tagged transporters. FE-L cells were mock transfected, transiently transfected with single human and Chinese hamster transporters fused with either GFP or BFP, or cotransfected with their combinations. Two days after the transfection, the cells were characterized by flow cytometry. **(A)** Gating strategy for flow cytometry characterization. The cells were washed by PBS, detached with trypsin-EDTA solution and transferred to 96-well plate with U-shaped bottom. The cellular suspension was pelleted by 3-minutes centrifugation with 450 × *g*. The pellets were resuspended in 50 μl of PBS without Hoechst and characterized by FACSymphony (BD) flow cytometer equipped with high throughput sampler. Cells were defined as follows: i. singlet events according to their ratio of area to height in forward scatter (FSC-A, FSC-H); ii. cells according to their area in forward scatter (FSC-A) and side scatter (SSC-A). From the resulting population, mean fluorescent intensities (MFI) of GFP (excitation 488 nm, emission 530/30 nm) and BFP (ex. 405 nm, em. 450/50 nm) were calculated. The population of transfected cells was characterized by fluorescence in iRFP channel (ex. 637 nm, em. 730/45 nm). The analyses were performed in FlowJo software (v10.10) and the frequency of parent (%) is shown. The transfected cells were further divided into four gates according to their expression of GFP and BFP. **(B)** A fraction of cells successfully transfected with plasmids encoding transporters. Transfected cells express the iRFP marker. **(C)** The MFI of GFP corresponds with the expression of transporters fused with GFP. **(D)** The MFI of BFP corresponds with the expression of transporters fused with BFP. **(E)** Fraction of transfected cells that express both transporters (turquoise with percentage value), GFP-tagged transporter only (green), BFP-tagged transporter only (blue) or no transporter (grey). In B-D, values are shown as means with relative standard deviation (RSD); in E, values are shown as means; total numbers of biological replicates are shown as n.

(A) Mock infection (fraction of mCherry-positive cells ±RSD)

BFP	mock-transfected	GFP: hsEAAT1	GFP: hsASCT1	GFP: hsASCT2	GFP: cgASCT1	GFP: cgASCT2
mock-transfected	0 ±0% (n = 9)					
single transfection		4.9e-5 ±283% (n = 9; *P* > 0.99)	2.6e-5 ±283% (n = 9; *P* > 0.99)	2.6e-5 ±283% (n = 9; *P* > 0.99)	1.6e-5 ±283% (n = 9; *P* > 0.99)	1.7e-5 ±283% (n = 9; *P* > 0.99)
hsEAAT1	1.1e-5 ±283% (n = 9; *P* > 0.99)	4.3e-5 ±283% (n = 9; *P* > 0.99)	1.4e-5 ±283% (n = 9; *P* > 0.99)	1.9e-5 ±283% (n = 9; *P* > 0.99)	2.7e-5 ±190% (n = 9; *P* = 0.85)	1.8e-5 ±283% (n = 9; *P* > 0.99)
hsASCT1	1.6e-5 ±283% (n = 9; *P* > 0.99)	0 ±0% (n = 9; *P* > 0.99)	0 ±0% (n = 9; *P* > 0.99)	0 ±0% (n = 9; *P* > 0.99)	7.3e-5 ±202% (n = 9; *P* = 0.47)	4.1e-5 ±283% (n = 9; *P* > 0.99)
hsASCT2	1.4e-5 ±224% (n = 6; *P* = 0.4)	3.3e-5 ±200% (n = 9; *P* = 0.47)	0 ±0% (n = 6; *P* > 0.99)	1.8e-5 ±283% (n = 9; *P* > 0.99)	0 ±0% (n = 9; *P* > 0.99)	0 ±0% (n = 9; *P* > 0.99)
cgASCT1	3.7e-5 ±200% (n = 9; *P* = 0.72)	0 ±0% (n = 9; *P* > 0.99)	1.7e-5 ±283% (n = 9; *P* > 0.99)	0 ±0% (n = 9; *P* > 0.99)	1.2e-4 ±159% (n = 9; *P* = 0.44)	3.1e-5 ±193% (n = 9; *P* = 0.72)
cgASCT2	2.8e-5 ±189% (n = 9; *P* = 0.72)	1.3e-5 ±283% (n = 9; *P* > 0.99)	0 ±0% (n = 9; *P* > 0.99)	1.4e-5 ±283% (n = 9; *P* > 0.99)	2.8e-5 ±192% (n = 9; *P* = 0.47)	7.7e-5 ±188% (n = 9; *P* = 0.72)

(B) Infection with Syncytin-1 (fraction of mCherry-positive cells ±RSD)

BFP	mock-transfected	GFP: hsEAAT1	GFP: hsASCT1	GFP: hsASCT2	GFP: cgASCT1	GFP: cgASCT2
mock-transfected	2.3e-4 ±201% (n = 9)					
single transfection		2.6e-5 ±187% (n = 9; *P* = 0.39)	3.1e-5 ±283% (n = 9; *P* = 0.12)	**0.11 ±23% (n = 9; *P* < 0.001)**	1.1e-5 ±283% (n = 9; *P* = 0.066)	4.5e-4 ±62% (n = 9; *P* = 0.075)
hsEAAT1	2.1e-5 ±283% (n = 9; *P* = 0.12)	1.1e-5 ±283% (n = 9; *P* = 0.066)	3.9e-5 ±192% (n = 9; *P* = 0.28)	**0.11 ±26% (n = 9; *P* < 0.001)**	6.8e-5 ±130% (n = 9; *P* = 0.89)	3.2e-4 ±98% (n = 9; *P* = 0.29)
hsASCT1	**0 ±0% (n = 9; *P* = 0.018)**	3.3e-5 ±192% (n = 9; *P* = 0.32)	3.3e-5 ±195% (n = 9; *P* = 0.28)	**9.0e-2 ±32% (n = 9; *P* < 0.001)**	1.7e-5 ±283% (n = 9; *P* = 0.084)	**4.4e-2 ±44% (n = 9; *P* < 0.001)**
hsASCT2	**0.16 ±11% (n = 6 ; *P* < 0.001)**	**9.9e-2 ±18% (n = 9; *P* < 0.001)**	**0.12 ±14% (n = 6 ; *P* < 0.001)**	**0.11 ±19% (n = 9; *P* < 0.001)**	**9.1e-2 ±32% (n = 9; *P* < 0.001)**	**8.2e-2 ±29% (n = 9; *P* < 0.001)**
cgASCT1	4.1e-5 ±187% (n = 9; *P* = 0.44)	1.4e-4 ±163% (n = 9; *P* = 0.84)	3.9e-5 ±199% (n = 9; *P* = 0.28)	**8.3e-2 ±38% (n = 9; *P* < 0.001)**	4.3e-5 ±196% (n = 9; *P* = 0.44)	**1.5e-3 ±60% (n = 9; *P* = 0.004)**
cgASCT2	**2.8e-3 ±55% (n = 9; *P* < 0.001)**	1.2e-3 ±74% (n = 9; *P* = 0.057)	**3.7e-2 ±39% (n = 9; *P* < 0.001)**	**0.10 ±24% (n = 9; *P* < 0.001)**	**7.9e-3 ±45% (n = 9; *P* < 0.001)**	**1.6e-3 ±42% (n = 9; *P* = 0.004)**

(C) Infection with BaEV (fraction of mCherry-positive cells ±RSD)

BFP	mock-transfected	GFP: hsEAAT1	GFP: hsASCT1	GFP: hsASCT2	GFP: cgASCT1	GFP: cgASCT2
mock-transfected	2.9e-5 ±203% (n = 9)					
single transfection		2.5e-5 ±191% (n = 9; *P* > 0.99)	1.6e-4 ±163% (n = 9; *P* = 0.68)	**7.7e-2 ±24% (n = 9; *P* < 0.001)**	4.9e-5 ±283% (n = 9; *P* > 0.99)	1.2e-5 ±283% (n = 9; *P* = 0.93)
hsEAAT1	8.8e-5 ±96% (n = 9; *P* = 0.25)	1.3e-4 ±236% (n = 9; *P* = 0.92)	2.5e-4 ±102% (n = 9; *P* = 0.18)	**7.9e-2 ±28% (n = 9; *P* < 0.001)**	1.4e-5 ±283% (n = 9; *P* = 0.89)	3.7e-5 ±283% (n = 9; *P* > 0.99)
hsASCT1	**1.5e-3 ±74% (n = 9; *P* = 0.002)**	**7.9e-4 ±65% (n = 9; *P* = 0.002)**	4.6e-4 ±137% (n = 9; *P* = 0.21)	**5.4e-2 ±34% (n = 9; *P* < 0.001)**	**5.5e-4 ±70% (n = 9; *P* = 0.012)**	**5.6e-4 ±77% (n = 9; *P* = 0.002)**
hsASCT2	**0.11 ±13% (n = 6; *P* < 0.001)**	**6.9e-2 ±18% (n = 9; *P* < 0.001)**	**6.6e-2 ±9% (n = 6; *P* < 0.001)**	**8.0e-2 ±22% (n = 9; *P* < 0.001)**	**6.3e-2 ±31% (n = 9; *P* < 0.001)**	**6.1e-2 ±30% (n = 9; *P* < 0.001)**
cgASCT1	2.8e-5 ±189% (n = 9; *P* > 0.99)	4.4e-5 ±187% (n = 9; *P* > 0.99)	1.9e-4 ±135% (n = 9; *P* = 0.28)	**5.8e-2 ±36% (n = 9; *P* < 0.001)**	2.2e-5 ±283% (n = 9; *P* = 0.74)	4.6e-5 ±203% (n = 9; *P* > 0.99)
cgASCT2	7.3e-5 ±202% (n = 9; *P* = 0.72)	2.9e-5 ±190% (n = 9; *P* > 0.99)	6.9e-5 ±149% (n = 9; *P* = 0.82)	**6.9e-2 ±29% (n = 9; *P* < 0.001)**	1.4e-4 ±116% (n = 9; *P* = 0.35)	2.7e-5 ±283% (n = 9; *P* > 0.99)

(D) Gating strategy: infection of cotransfected cells

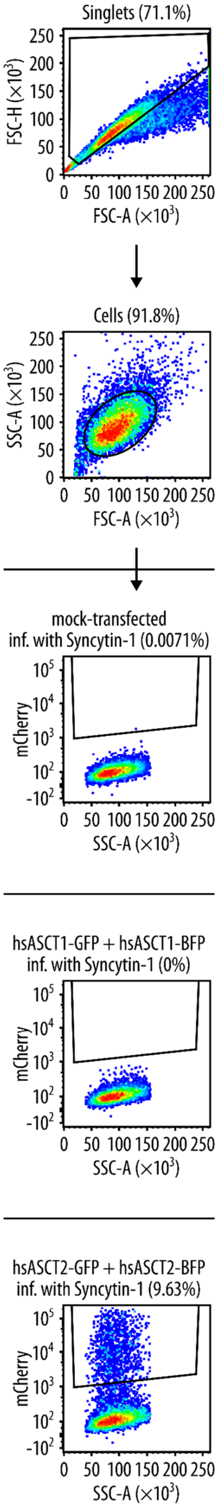

Fig. S11. Sensitivity of cells transiently transfected with combinations of human or Chinese hamster transporters. Two days after transfection, the cells were infected with MOI 3 or mock infected and the infection was quantified by flow cytometry. At the time of the infection, the expression of transporters was assessed (Fig. S10). **(A-C)** Results of mock-infection **(A)**, infection with Syncytin-1 pseudotype **(B)**, and BaEV pseudotype **(C)**. The fractions of infected cells are shown as means with relative standard deviation (RSD). **(D)** Gating strategy for infection quantification. The cells were detached with trypsin, fixed in paraformaldehyde (1% final concentration), transferred to 96-well plate with U-shaped bottom and characterized by FACSymphony (BD) flow cytometer equipped with high throughput sampler. Infected cells were defined as follows: i. singlet events according to their ratio of area to height in forward scatter (FSC-A, FSC-H); ii. cells according to their area in forward scatter (FSC-A) and side scatter (SSC-A); iii. infected cells according to their signal in mCherry channel (ex. 561 nm, em. 610/20 nm). The analyses were performed in FlowJo software (v10.10) and the frequency of parent (%) is shown. The experiment was repeated three times and total numbers of biological replicates are shown as n. *P* values from mock-transfected cells were calculated with Mann-Whitney non-parametric test with Holm-Šídák correction for multiple comparisons. *P* values lower than 0.05 are in bold.

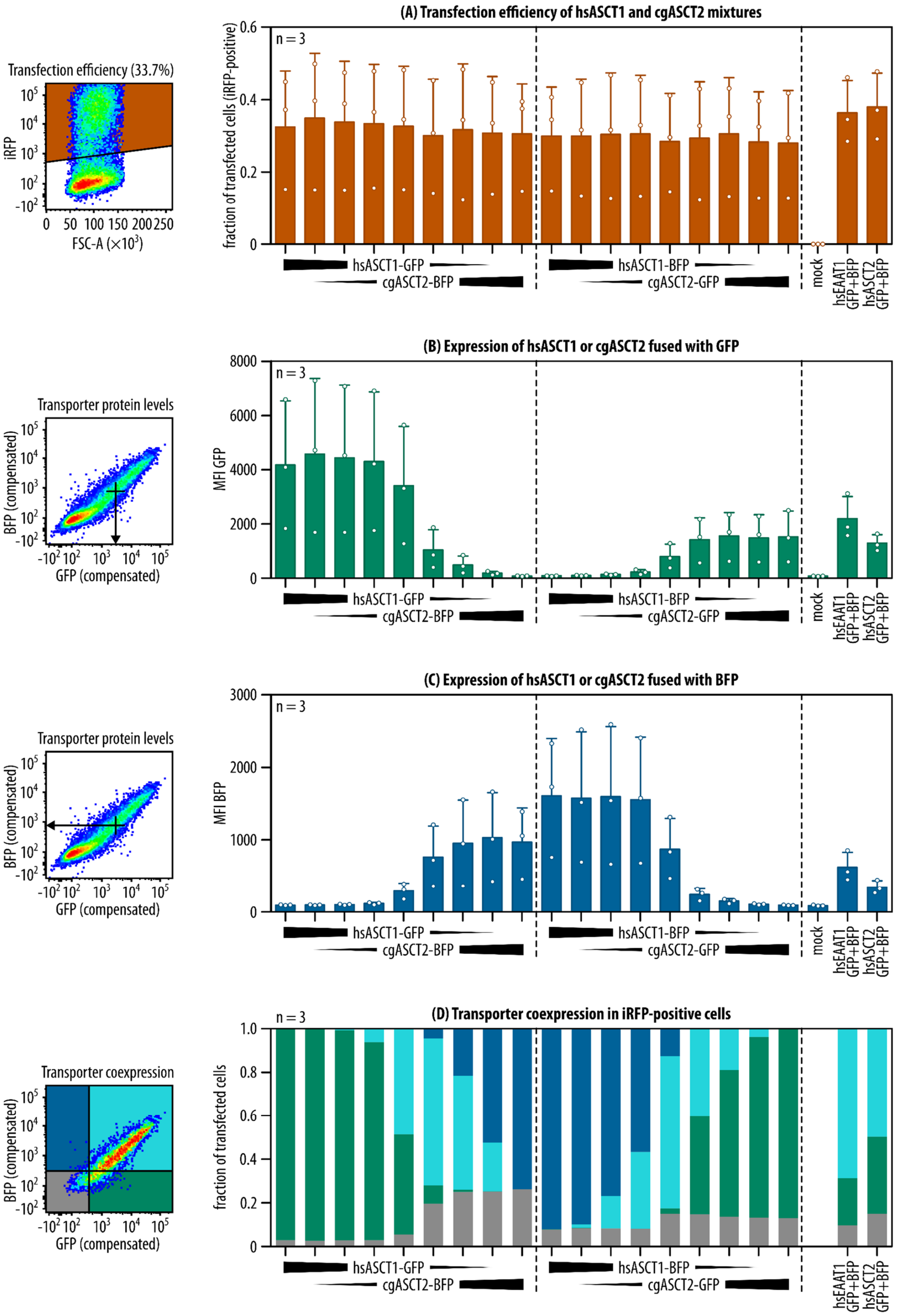

(A) Transfection efficiency of hsASCT1 and cgASCT2 mixtures
Transfection efficiency (33.7%)
iRFP
FSC-A (×10³)
fraction of transfected cells (iRFP-positive)
n = 3
hsASCT1-GFP
cgASCT2-BFP
hsASCT1-BFP
cgASCT2-GFP
mock
hsEAAT1 GFP+BFP
hsASCT2 GFP+BFP
(B) Expression of hsASCT1 or cgASCT2 fused with GFP
Transporter protein levels
BFP (compensated)
GFP (compensated)
MFI GFP
(C) Expression of hsASCT1 or cgASCT2 fused with BFP
MFI BFP
(D) Transporter coexpression in iRFP-positive cells
Transporter coexpression
fraction of transfected cells

Fig. S12. Flow cytometry analysis of transient expression of transporters in cells from Figure 4C. FE-L cells were mock transfected or transiently transfected with human ASCT1 and Chinese hamster ASCT2 fused with either GFP or BFP in ratios of 100:0, 99:1, 96:4, 90:10, 50:50, 10:90, 4:96, 1:99, 0:100. Two days after the transfection, the cells were characterized by flow cytometry as in Fig. S10A. **(A)** Fraction of cells successfully transfected with plasmids encoding transporters. Transfected cells express iRFP marker. **(B)** Mean fluorescence intensity of GFP corresponds with expression of transporters fused with GFP. **(C)** Mean fluorescence intensity of BFP corresponds with expression of transporters fused with BFP. **(D)** Fraction of transfected cells that express both transporters (turquoise), GFP-tagged transporter only (green), BFP-tagged transporter only (blue) or no transporter (grey). Total numbers of biological replicates are shown as n. The data in A-C represents average values with standard deviation. The data in D represents average values. Mock, non-transfected cells.

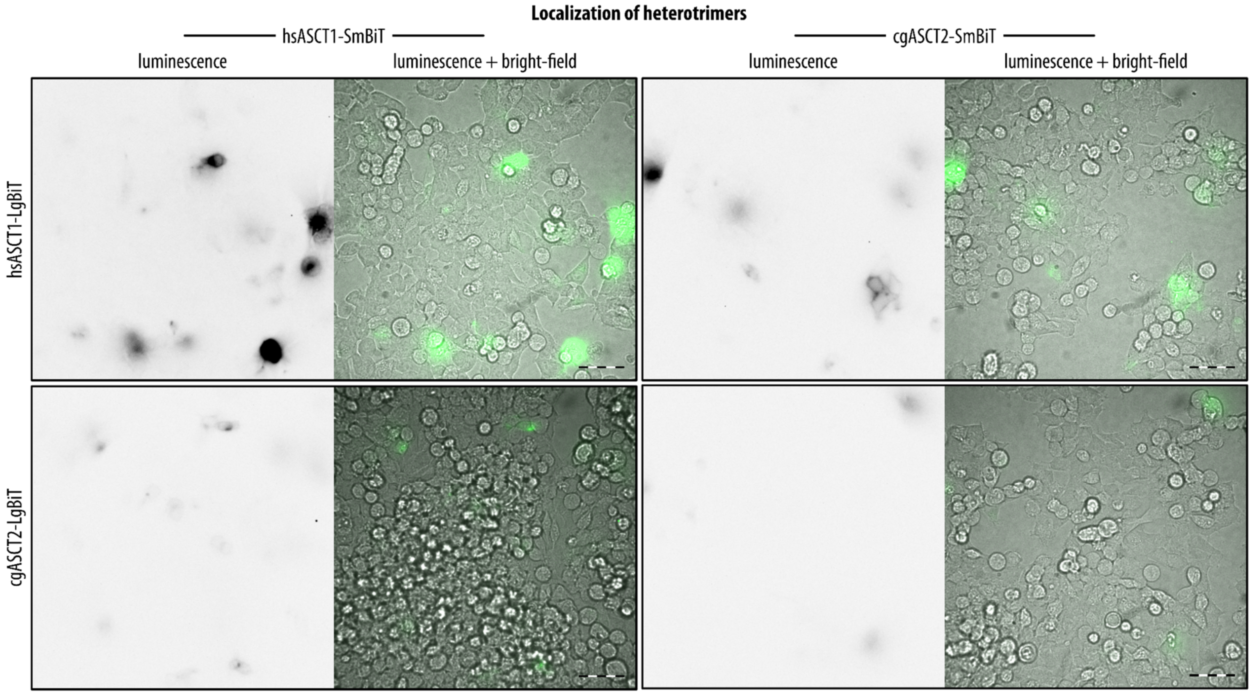

Fig. S13. Luminescence microscopy of receptor trimers. FE cells were transiently cotransfected with homologous or heterologous combinations of human and Chinese hamster ASCT1 or ASCT2 fused either with LgBiT or SmBiT (1:1 ratio for LgBiT:SmBiT). Luciferase activity was visualized by the addition of Nano-Glo Live Cell Reagent. Images were immediately captured using an Olympus LV200 bioluminescence microscope. Scale bar 50 µm.

Table S1. Parameters of mass spectrometry analysis.

Compound ID	Q1 mass	Q3 mass	Dwell time (ms)	DP (V)	EP (V)	CE (V)	CXP (V)
d3-serine	109.1	63.1	25	35	10	16	8
d5-glutamine	152.1	88.0	25	50	10	25	8
d7-arginine	182.2	60.1	25	35	10	21	8

SI References

1. J.-P. Concordet, M. Haeussler, CRISPOR: intuitive guide selection for CRISPR/Cas9 genome editing experiments and screens. *Nucleic Acids Res.* **46**, W242–W245 (2018).
2. T. Masuda, M. Tomita, Y. Ishihama, Phase Transfer Surfactant-Aided Trypsin Digestion for Membrane Proteome Analysis. *J. Proteome Res.* **7**, 731–740 (2008).
3. R. Vento-Tormo, *et al.*, Single-cell reconstruction of the early maternal–fetal interface in humans. *Nature* **563**, 347–353 (2018).
4. C. Megill, *et al.*, cellxgene: a performant, scalable exploration platform for high dimensional sparse matrices. *bioRxiv*, 2021.04.05.438318 (2021). https://doi.org/10.1101/2021.04.05.438318.
